# Is women's empowerment a pathway to improving child nutrition outcomes in a nutrition-sensitive agriculture program?: Evidence from a randomized controlled trial in Burkina Faso

**DOI:** 10.1016/j.socscimed.2019.05.016

**Published:** 2019-07

**Authors:** Jessica Heckert, Deanna K. Olney, Marie T. Ruel

**Affiliations:** Poverty, Health, and Nutrition Division, International Food Policy Research Institute, 1201 I Street, NW, Washington, DC, 20005, USA

**Keywords:** Women's empowerment, Child nutrition, Mediation analysis, Agriculture, Nutrition-sensitive programs, Burkina Faso

## Abstract

Nutrition-sensitive programs in low- and middle-income countries often aim to improve child nutrition outcomes in part by empowering women. Although previous studies have found cross-sectional associations linking women's empowerment and child nutritional status, there is limited empirical evidence supporting the hypothesis that empowering women as part of an intervention will, in turn, improve child nutritional outcomes. We tested this hypothesis using two waves of data from a cluster-randomized controlled trial of a nutrition-sensitive agricultural program in Burkina Faso. With structural equation models, we examined whether four domains of women's empowerment—purchasing decisions, healthcare decisions, family planning decisions, and spousal communication—mediated the program's impact on reducing wasting and increasing hemoglobin among children who were three to 12 months old at the start of the two-year program. We found that improvements in women's empowerment in the domains of spousal communication, purchasing decisions, healthcare decisions, and family planning decisions contributed to the program's impact on reducing wasting with the largest share being attributable to spousal communication. Improvements in women's empowerment did not contribute to the increase in hemoglobin. These findings provide the first evidence from a randomized controlled trial that women's empowerment is a pathway by which a nutrition-sensitive program can improve child wasting. Programs that aim to improve child nutritional status should incorporate interventions designed to empower women.

## Highlights

• A nutrition- and gender-sensitive agriculture program was evaluated in Burkina Faso.
• The program reduced child wasting and improved women's empowerment (WE).
• Reductions in child wasting were partially mediated by improvements in WE.
• This is the first experimental evidence that increasing WE improves child nutrition.
• Programs to reduce wasting can be more effective if they also target WE.

## 1 Introduction

Nutrition-sensitive programs address the underlying determinants of malnutrition and incorporate both nutrition goals and interventions (Ruel and Alderman, 2013). Such programs, which may include agricultural, social safety net, early child development, education, water and sanitation, and/or health and family planning interventions in low- and middle-income countries, often target mothers as primary caregivers and incorporate components that empower women to exert agency and make key decisions. The justification for empowering women as part of nutrition-sensitive programs is supported by a growing body of empirical evidence that concludes that empowered mothers have children with better nutritional outcomes (Begin et al., 1999; Franckel and Lalou, 2009; Hindin, 2000; Malapit et al., 2015; Malapit and Quisumbing, 2015; M. Shroff, Griffiths, Adair, Suchindran and Bentley, 2009; M. R. Shroff et al., 2011). However, these studies primarily draw conclusions from association studies using cross-sectional data and fail to provide evidence on whether empowering women leads to improvements in their children's nutritional status (Cunningham et al., 2014; van den Bold et al., 2013).

Herein, we examine the relationship between women's empowerment and child nutritional status using data from two waves of a cluster-randomized controlled trial of the Enhanced Homestead Food Production (E-HFP) program, a nutrition- and gender-sensitive agriculture program implemented by Helen Keller International in Burkina Faso. The E-HFP program aimed to improve children's nutritional status, specifically reduce stunting (height-for-age Z score (HAZ) < −2 SD, a marker of chronic undernutrition) and wasting (weight-for-height Z score (WHZ) <-2 SD, an indicator of acute undernutrition) and increase hemoglobin concentration (Hb). It was designed to do so through the provision of agricultural assets and delivery of a behavior change communication (BCC) strategy focused on agricultural activities and optimal infant and young child feeding (IYCF), health, hygiene, and care practices. Together these program inputs were expected to improve children's nutritional status by increasing women's production of nutrient-rich foods, children's intake of these foods, and women's income and control over resources through sale of surplus production. The program was also expected to serve as a basis for women's empowerment by increasing access to program inputs, control over the associated resources, and maternal knowledge of and ability to implement optimal agriculture, nutrition, and health practices. In turn, it was expected these improvements would better position women to negotiate the allocation of resources within their households in favor of the health and nutrition of themselves and their children. Previous findings showed that the E-HFP program increased women's empowerment and control over resources, reduced wasting (but not stunting), and increased Hb among children (Olney et al., 2016; Olney et al., 2015; van den Bold et al., 2015). These studies, however, did not specifically test the pathways through which child nutrition improved.

Our first objective is to examine the association between women's empowerment—four domains (purchasing decisions, healthcare decisions, family planning decisions, and spousal communication) and an aggregate score—and child wasting and Hb using baseline data and test whether these effects are moderated by household economic status. This also allows us to establish comparability between this sample and previous studies. We then continue with our principal objective, which is to examine the extent to which the E-HFP program's positive impact on child wasting and Hb is attributable to its success in empowering women. We consider the same four domains of women's empowerment and test whether improvements in these domains mediated improvements in child nutritional status using structural equation models (SEM). This paper expands current work on the complex relationship between women's empowerment and child nutrition by considering the multidimensionality of women's empowerment, examining the role of women's empowerment as a tool for leveraging resources in the household, and determining whether the improvements in child nutritional status from the E-HFP program were due, at least in part, to its success in empowering women.

### 1.1 Background

Women's empowerment is the ability to make strategic choices and exercise agency (Kabeer, 2005). Restrictions on women's agency in the domestic sphere—including spousal relationships, access to resources, and input into health decisions—is a key factor linking the availability of physical and human resources to the adoption of optimal childcare practices and improved nutritional status among children (Shroff et al., 2009). We conceptualize empowerment as distinct from women's status, which includes characteristics such as educational attainment. Though women's status may serve as a resource for empowerment, these characteristics are relatively fixed in adulthood. Empowerment, however, is still malleable, and women may be empowered or disempowered through various experiences (Kabeer, 2005), including participation in a nutrition- and gender-sensitive agriculture program such as the E-HFP program.

Empowerment is important not only because of its intrinsic value, but also the instrumental value it provides in enabling the use of available resources (Kabeer, 2005). This implies, first, that the relationship between empowerment and better child health and nutrition status may be moderated by resource availability (including physical, economic, and human resources) (i.e., effect modification). However, we found no examples that explicitly tested this hypothesis. Additionally, it implies that an intervention, such as a nutrition-sensitive program that empowers women and equips them with a range of resources, women's empowerment may be one means by which the program improves child nutrition outcomes. A number of nutrition-sensitive programs, such as those that incorporate resource transfers, facilitate microcredit, and empower women through social support and rights-based approaches hypothesize that it is a pathway, (Gillespie and Hodge, 2016; Jalal and Frongillo, 2013), but we found no evidence confirming the importance of this pathway in achieving nutritional impacts.

Empowerment is a multidimensional construct, and women who are empowered in some domains may not be in others (Alkire et al., 2013; Kabeer, 2005). Importantly, different domains may influence child nutritional status through a range of mechanisms. One often explored link is between child nutritional status and women's input into economic decision making, such as purchasing decisions or economic production. Economic empowerment may help women prioritize the purchase of specific foods, hygiene products, and medications. Women's control over economic production decisions may be related to food production for home consumption (Perry, 2005), and empowerment in agricultural production is associated with better child feeding practices (Malapit and Quisumbing, 2015).

Distinct from economic decisions, contributions to other decisions may also contribute to better child health. Several studies link maternal decision-making to better child nutritional status—most using a general household decision-making indicator based on items in the Demographic and Health Surveys. Evidence finds that children of mothers who participated in more household decisions were less likely to be wasted and stunted (India) (Shroff et al., 2011), more likely to seek antenatal care and deliver with a skilled attendant (multi-country) (Ahmed et al., 2010), more likely to use antenatal and postnatal care (India) (Mistry et al., 2009), and more likely to have fully vaccinated children (Ethiopia) (Ebot, 2015).

Although general decision-making indicators are common, women may have different amounts of decision making input in different aspects of household life, and few studies have focused on specific areas. For decisions related to child feeding in rural Chad, mothers’ input was associated with higher HAZ for children 12-71-month-olds (Begin et al., 1999). Women with input into healthcare decisions were more likely to use antenatal and postnatal care and were better able to seek care for and treat sick children (Sado et al., 2014). Moreover, the ability to make family planning decisions may improve child nutritional outcomes by helping mothers optimize birth timing and achieve desired family size. High fertility and short birth intervals may influence child nutritional status (both existing and future ones) by increasing the degree to which resources are diluted among children (Desai, 1995; Öberg, 2015) and facilitating disease transmission among siblings (Conde-Agudelo et al., 2012). The nutritional status of breastfed children is hindered if children are weaned early to allow for conception (Fledderjohann et al., 2014) or suboptimal breastfeeding occurs following conception (Conde-Agudelo et al., 2012). These decisions may also influence children conceived following short birth intervals due to maternal nutritional depletion (Conde-Agudelo et al., 2012).

Moreover, a women's ability to communicate about her children's nutrition and health needs with her husband, and negotiate to prioritize these needs, may also be related to child health. We identified no previous studies that linked this factor to child nutritional status, though it was found to mediate the positive association between women's education and dietary diversity in Bangladesh (Sinharoy et al., 2018). Additionally, an intervention that aimed to increase father involvement and improve spousal communication in Rwanda had an impact on care practices, which may improve nutritional outcomes (Doyle et al., 2018). In Burkina Faso, as in other neighboring countries, mothers generally fulfill caregiver roles and manage children's daily activities. Meanwhile, fathers maintain moral and economic authority over healthcare, determining when and what type of antenatal (Somé et al., 2013) and curative care is sought (Franckel and Lalou, 2009). Thus, when women can express their needs to their husbands, it may enable better access to inputs, and better health and nutritional status.

Based on the current literature, we used data from an experimental evaluation to test three hypotheses. First, using only baseline data, we tested whether women's empowerment was associated with a lower prevalence of wasting and higher Hb. We examined this relationship using four separate domains (purchasing decisions, spousal communication, healthcare decisions, and family planning decisions) and an aggregate empowerment score. Second, to test the hypothesis that women's empowerment operates by leveraging available resources, particularly in a low resource setting, we tested for an interaction between empowerment and household economic status. Third, using baseline and follow-up data, we tested the hypothesis that the E-HFP program's impact on child wasting and Hb was, at least in part, mediated by increases in women's empowerment.

## 2 Methods

### 2.1 Study setting

The E-HFP program was designed and implemented in Fada N'Gourma in eastern Burkina Faso. Burkina Faso has a high prevalence of child undernutrition caused by inadequate intake of nutrient-rich foods, repeated illnesses, and suboptimal care and feeding practices (Olney et al., 2015). Among Burkinabé children younger than 5, 88% are anemic—among the worst in the world (Institut National de la Statistique et de la Démographie and ICF International, 2012). Additionally, the prevalence of wasting (16%) and stunting (35%) are both high (Institut National de la Statistique et de la Démographie and ICF International, 2012). In the region where this study takes place, child nutritional outcomes are worse than the country as a whole with 91% anemic, 18% wasted, and 43% stunted (Institut National de la Statistique et de la Démographie and ICF International, 2012).

### 2.2 Program description and evaluation design

The primary program objective was to improve maternal and child nutrition, and an intermediate goal was to empower women. The program targeted mothers of children aged 3–12 months at the start of the program and included 3 main components: 1) agricultural training and inputs (e.g., tools, seeds, chickens) targeted to women to promote small-scale agriculture and increase production of nutrient-rich foods (e.g., eggs, vegetables) for sale and consumption; 2) BCC programming to foster the adoption of optimal health and nutrition practices; and 3) community-focused activities to develop land-use agreements to promote women's access to land for agricultural use. It was expected that these interventions would form the basis of skills and knowledge to empower women and would increase their self-efficacy, control over household resources, input into decisions, ability to negotiate with household members in favor of nutrition-related actions, and respect in the community.

For the cluster-randomized controlled trial, villages were randomly assigned to a control arm (n = 25) or one of two treatment arms, which differed in who implemented the BCC strategy—either older women leaders (n = 15) or health committee members (n = 15) (Olney et al., 2015). The two treatment arms were pooled for these analyses, because they implemented identical BCC curriculums and there were no differential impacts on women's empowerment based on who implemented it.

Household surveys were used to collect data at baseline (February–May 2010) and from the same mothers and children two years later (February–June 2012). Both waves included household demographic and socio-economic characteristics, maternal and child anthropometry (measured by trained anthropometers), child Hb (blood collected by fingerpick), and women's empowerment (See Olney et al., 2015 and Olney et al., 2016 for complete details.). Informed consent was obtained from the household head or the mother of the child, and the protocol was approved by the Ministry of Health of Burkina Faso, and the institutional review board of the International Food Policy Research Institute.

### 2.3 Variables

Our analysis focused on two indicators of child nutritional status for which the E-HFP program had statistically significant impacts: wasting and Hb. There were no program impacts on stunting, HAZ, underweight, or WAZ. We used wasting (binary) to focus on shifts in the lower end of the WHZ distribution. Because of the high prevalence of anemia (89%) in the sample, the continuous variable (Hb) was better suited for the analyses.

In previous analysis of household decisions and spousal communication data, exploratory factor analysis (EFA), informed by theoretical justifications, was used to identify seven domains related to women's empowerment (Olney et al., 2016). Five were relevant to our current study: spousal communication, purchasing decisions, healthcare decisions, family planning decisions, and IYCF decisions. IYCF decisions were not included, because nearly all mothers reported that they contributed to them. (We excluded meeting with other women and social support. Both may help empower women, but do not measure it directly, and meeting with other women could not be disentangled from program participation in this case). Spousal communication items included how often the respondent spoke to her spouse about: professional or agricultural activities, domestic activities, expenses, community events, her child's health, her child's food, and her own health (coded never = 0, sometimes = 1, often = 2). Purchasing decisions included whether she could make the decisions to purchase small quantities of food, larger quantities of food, clothing for herself, medication for herself, and special foods for her children. Healthcare decisions included whether she contributed to decisions to consult a doctor when she was pregnant and what to do when a child was sick. Family planning decisions included whether she contributed to decisions to use a contraceptive method and have another child. Respondents reported which household members contributed to the decisions, and responses were recoded 0/1 for whether she contributed either solely or jointly. Supplementary Table 1 reports EFA results for items related to the four domains included in this paper; no item loaded on multiple factors. The third factor was split into family planning decisions and healthcare decisions based on the theoretical justification that women experience different limitations in these areas, although two items for each domain is not ideal. A confirmatory factor analysis (CFA) model that included all four factors was then fit for each wave of data. Supplementary Table 2 shows that items loaded significantly on their respective latent factors and fit statistics were adequate. We used different approaches to incorporate these items into the cross-sectional and mediation analysis, and details are in the corresponding sections.

Household economic status was measured using a standardized factor score that was constructed based on a principal components analysis of five housing and utility characteristics, which were coded 0 for poor quality and 1 for higher quality (Filmer and Pritchett, 2001). These included flooring material (dirt vs. improved), roofing material (straw vs. improved), wall material (non-improved vs. block), having a latrine, and having electricity.

The household roster provided information on child sex and age; maternal age, height, and whether she had ever attended school; the size of the household; whether it was polygynous, and whether the head had ever attended school.

### 2.4 Analytic sample

For both the cross-sectional and mediation analysis, the samples were limited to mother-child dyads in which the mother lived with a spouse. This restriction ensured the interpretability of the women's empowerment questions, which sometimes assumed that a husband was part of the household. Marriage is nearly universal in Burkina Faso, and this restriction reduced the sample size from 1265 to 1058 for the cross-sectional analysis and 1035 for the mediation analysis.

Households that were excluded had 1.8 fewer members, were less likely to be polygynous, and included mothers that were 1.7. They did not differ significantly on household economic status, whether they had attended school, or the age, sex, and nutritional status of their child (i.e., anemic, Hb, wasting, WHZ) at baseline.

### 2.5 Cross-sectional analysis of baseline data

To examine the cross-sectional associations between child nutritional status (wasting and Hb) and women's empowerment (four domains: spousal communication, purchasing decisions, healthcare decisions, and family planning decisions and an aggregate score), we first constructed women's empowerment variables. The CFA models were used to predict standardized factor scores for each latent variable, which reflect respondents' spacing relative to one another on the latent variable. We then constructed an aggregate women's empowerment score in which the four latent domains loaded on a fifth, higher level, latent variable.

We divided the sample into two age groups: 1) 3–5 months and 2) 6–12 months, which was motivated by differences in the drivers of nutritional status and recommended feeding practices at these different ages. Six months marks the recommended age to introduce complementary foods in addition to breastmilk and when infant mobility increases (Kramer and Kakuma, 2004). These changes increase the nutritional needs for infants and exposes them to a wider range of pathogens.

We used multivariate logistic regression to predict wasting and linear regression to predict Hb. In the first set of models, for each of the two dependent variables, we separately included factor scores for the four domains of empowerment and aggregate empowerment. We recognize that using factor scores ignores the error structure associated with the latent variable. However, this approach allows us to include an interaction between each domain of empowerment and household economic status in the following model. All models controlled for potential confounding factors, including child gender and age; maternal age, height (when wasting was the dependent variable), and whether she ever attended school; household economic status and size; whether the household head ever attended school; and whether the household was polygynous.

In the second set of models, we included an interaction between empowerment and household economic status to test the hypothesis that women's empowerment operates by leveraging available resources. List-wise deletion was used for these analyses, and 40 additional cases were lost because of missing anthropometric data. All models accounted for clustering, and coefficients were considered significant if p < 0.05 and marginally significant if p < 0.10. Regression analyses were conducted using Stata 14.2.

### 2.6 Mediation analysis

Using SEM, we examined whether changes in women's empowerment partially mediated the impact of the E-HFP program on reducing wasting and increasing Hb among children 3–12 months old at baseline. The dependent variables were Δwasting and ΔHb, each a simple difference (T_2_-T_1_). Using the change score as the dependent variable in SEM is equivalent to difference-in-difference regression estimates, which were used to estimate program impact (Olney et al., 2015).

**Fig. 1:**
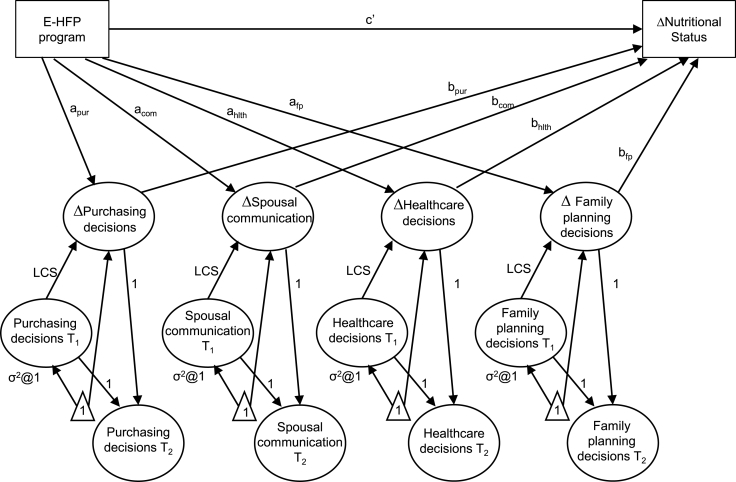
Structural equation model depicting change in women's empowerment as a mediating pathway between the program and change in nutritional status. Note: Each domain of empowerment is a latent variable measured by multiple indicators, which, along with error terms, are not shown for simplicity. Observed variables are depicted by squares, latent variables by circles, and constants by triangles. Consistent with the latent change score approach, the variance of each T_1_ latent variable, paths from T_1_ to T_2,_ and paths from Δlatent variable to T_2_ are held constant at one. E-HFP = Enhanced Homestead Food Production. LCS = latent change score.

To incorporate latent variables for the four empowerment domains at two time points, CFA models were fit for both time points using the same factor structure from the previous step. A common factor structure with similar loadings was present for both. To include a variable representing change on each (latent) domain of empowerment, we constructed a latent change score (LCS) for each of the four domains (Fig. 1). Given a common factor structure across time points, LCSs makes it possible to incorporate change in a latent variable into SEM, which cannot be done directly while preserving the measurement structure (McArdle and Nesselroade, 2014). In using LCSs we mimic a change score (EmpowermentT_2_ = 1* EmpowermentT_1_ + 1*Δempowerment) where the unobserved Δempowerment is defined as the part of EmpowermentT_2_ that differs from EmpowermentT_1_ (McArdle and Nesselroade, 2014).

We assessed mediation by calculating the indirect effect (i.e., portion of the total effect attributable to the mediator) of E-HFP on the change in nutritional status that was attributable to each hypothesized mediator (Δempowerment). Each of the four hypothesized mediators was regressed on the independent variable (treatment) to estimate the values of the four *a* paths in Fig. 1. In a fifth equation the dependent variable (e.g., Δwasting) was regressed on the four mediators (to estimate the four *b* paths in Fig. 1) and the independent variable (to estimate the *c’* path in Fig. 1) (Preacher, 2015). For each hypothesized mediator the indirect effect was calculated from the values of the respective *a* and *b* paths using bootstrapping (1000 repetitions) to account for concerns that the product a*b is often non-normally distributed (Preacher and Hayes, 2008). The bootstrapping procedure often produces confidence intervals that are asymmetric, and we based statistical inference on whether confidence intervals overlapped zero; 95% confidence intervals were significant, and 90% confidence intervals marginally significant.

As we are interested in the joint contribution of the four domains of women's empowerment (not whether one domain is more influential than another), we tested a model that simultaneously included all four domains in which the covariances between each pair of the four mediators are freely estimated. The total indirect effect of empowerment is the sum of the indirect effects of the four domains. The remaining effect of the independent variable after accounting for the mediator (path *c’* in Fig. 1) is called the direct effect. The total effect is the sum of the direct and indirect effects, which is also the value of the dependent variable regressed on the independent variable without including any hypothesized mediator (path *c,* not shown in Fig. 1).

Using this approach, we fit models for Δwasting and ΔHb. The equations predicting the impact of the program on each empowerment domain also control for child sex and age; maternal age, height, and education; household economic status; and household head's education. The equations predicting each dependent variable control for child sex and age and maternal age and height (except for ΔHb).

Full information maximum likelihood was used to account for attrition and other missing data. We evaluated model fit based on the root mean square error of approximation (RMSEA), comparative fit index (CFI), and the Tucker-Lewis index (TLI). Cut-off values for the RMSEA (range 0–1) are <0.05 for a good fit and <0.08 for an adequate fit. For the CFI and TLI (range 0–1), they are >0.95, or >0.90 under less restrictive guidelines. SEM models were fit in R (3.3.2) using the lavaan package (0.5–22) (Rosseel, 2012).

## 3 Results

### 3.1 Descriptive characteristics

In the samples for both the cross-sectional (Table 1) and the mediation analyses (Table 2), approximately 26% were stunted, and the average Hb was 9.0 g/dL at baseline. The samples were evenly split by gender, and children were on average 7.3 months old at baseline. Mothers were on average 28 years old, 161 cm tall, and less than 7% had ever attended school. Approximately 10% of household heads had ever attended school. For the additional characteristics used in the cross-sectional analysis, households, on average, had eight members, and a little less than half were polygynous. The average change between baseline and follow-up was negative for wasting and positive for Hb, including the control group, which is consistent with early development in nutritionally at-risk populations. In the sample used for the mediation analysis, baseline characteristics did not differ significantly (p < 0.05) between the study arms.

**Table 1:** Characteristics of sample for cross-sectional analysis.

n =	Total		3-5-month-olds		6-12-month-olds	
	1058		404		654	
	Mean/%	(se)	Mean/%	(se)	Mean/%	(se)
**Nutritional status**						
Wasting	26.5%		25.9%		26.9%	
Hemoglobin	9.0	(1.7)	9.1	(1.7)	8.9	(1.7)
**Child characteristics**						
Male	50.3%		50.0%		50.5%	
Age (months)	7.3	(2.6)	4.6	(.8)	9.0	(1.8)
**Maternal characteristics**						
Age (years)	28.2	(6.4)	28.0	(6.2)	28.4	(6.5)
Height (cm)	161.2	(6.2)	161.3	(6.2)	161.1	(6.2)
Attended school	6.7%		8.4%		5.7%	
**Household characteristics**						
Head attended school	10.4%		11.9%		9.5%	
Economic status factor score	0.0	(1.2)	0.0	(1.3)	−0.1	(1.2)
Household size	8.1	(3.8)	8.3	(3.9)	8.0	(3.7)
Polygynous	45.6%		45.8%		45.4%	
**Women's empowerment (standardized factor score)**						
Purchasing decisions	0.00	(.22)	0.00	(.22)	0.00	(.22)
Spousal communication	0.00	(.46)	0.02	(.45)	−0.01	(.47)
Healthcare decisions	0.00	(.37)	0.02	(.37)	0.00	(.37)
Family planning decisions	0.00	(.48)	0.00	(.48)	0.00	(.48)
Aggregate empowerment	0.00	(.33)	0.00	(.33)	0.00	(.32)

**Table 2:** Characteristics of sample for longitudinal mediation models.

n =	Total sample		Control		Treatment		Difference
	1035		425		610		by arms
	Mean/%	(se)	Mean/%	(se)	Mean/%	(se)	p-value
**Nutritional status at baseline**							
Wasting	26.1%		24.4%		27.3%		.327
Hemoglobin (g/dL)	9.0	(1.7)	9.1	(1.6)	8.9	(1.7)	.010
**Change in nutritional status**							
Δwasting	-.17	(.52)	-.13	(.53)	-.20	(.51)	.060
Δhemoglobin	.75	(2.17)	.62	(2.03)	.85	(2.25)	.104
**Child characteristics**							
Male	50.7%		52.5%		49.5%		.349
Age T_1_ (months)	7.3	(2.8)	7.4	(2.8)	7.3	(2.7)	.581
**Maternal characteristics**							
Age T_1_ (years)	28.4	(6.4)	28.8	(6.5)	28.2	(6.3)	.142
Height (cm)	161.3	(6.3)	161.4	(6.2)	161.2	(6.3)	.647
Attended school	6.5%		6.1%		6.8%		.674
**Household characteristics**							
Head attended school	10.7%		11.2%		10.4%		.686
Economic status factor score	.0	(1.2)	.1	(1.3)	-.1	(1.2)	.076

The empowerment scores used in the cross-sectional analyses were standardized to have a mean of zero, and the correlations among the items were consistent with the presence of four distinct factors (Supplementary table 3). The correlations for the same items across time were positive, but weak.

### 3.2 Cross-sectional analysis of baseline data to examine associations between women's empowerment and wasting and Hb

Among 3-5-month-olds, Model 1 found no significant main effects between children's wasting or Hb and women's empowerment in any of the four domains or the aggregate score (Table 3). Among 6-12-month-olds, we found that wasting was less prevalent when mothers had more input into purchasing decisions (β = −0.790, p < 0.10) (marginally significant) and when aggregate empowerment scores were higher (β = −0.552, p < 0.05). No other domains were significantly associated with wasting. In the case of Hb among 6-12-month-olds, findings revealed a marginally significant negative association with spousal communication (β = −0.330, p < 0.10) and a significant negative association with family planning decisions (β = −0.169, p < 0.05)—both opposite from what was hypothesized. Hb was not significantly associated with any of the other domains of empowerment.

**Table 3:** Results of cross-sectional multivariate regression analyses predicting wasting (logistic) and hemoglobin concentration (linear).

Age group	3-5-month-olds				6-12-month-olds			
Nutritional status outcome	Wasting		Hemoglobin (g/dL)		Wasting		Hemoglobin (g/dL)	
n =	Model 1	Model 2	Model 1	Model 1	Model 1	Model 2	Model 1	Model 2
	386	386	404	404	643	643	654	654
	β(se)	β(se)	β(se)	β(se)	β(se)	β(se)	β(se)	β(se)
Purchasing decisions	.535	.426	.155	.144	-.790†	-.837*	-.265	-.265
	(.579)	(.557)	(.345)	(.337)	(.422)	(.415)	(.313)	(.320)
Economic status	-.083	-.038	.000	.005	-.043	-.048	.032	.032
	(.101)	(.104)	(.059)	(.063)	(.072)	(.075)	(.173)	(.052)
Purchasing*economic		-.642*		-.086		-.353		.002
		(.315)		(.283)		(.311)		(.294)
Wald χ^2^	12.1	17.6			15.6	16.9		
F-statistic			6.5	.8			2.9	2.7
p = a	.208	.062	.535	.599	.075	.078	.007	.010

Spousal communication	.047	.025	.015	.014	-.114	-.081	-.330†	-.327†
	(.237)	(.238)	(.168)	(.169)	(.224)	(.222)	(.171)	(.173)
Economic status	-.073	-.072	.003	.003	-.035	-.041	.045	.045
	(.099)	(.099)	(.060)	(.061)	(.071)	(.067)	(.055)	(.056)
Communication*economic		-.200		-.025		.171		.018
		(.182)		(.148)		(.169)		(.139)
Wald χ^2^	10.5	11.2			13.6	15.3		
F-statistic			.9	.9			3.7	3.4
p = a	.313	.345	.532	.548	.139	.122	.001	.002

Healthcare decisions	.342	.330	-.356	-.350	-.161	-.173	.032	.035
	(.340)	(.339)	(.230)	(.229)	(.221)	(.232)	(.197)	(.205)
Economic status	-.071	-.060	.001	.006	-.039	-.036	.032	.031
	(.100)	(.099)	(.058)	(.057)	(.071)	(.074)	(.053)	(.052)
Healthcare*economic		-.330†		-.149		-.072		.031
		(.181)		(.149)		(.231)		(.169)
Wald χ^2^	11.6	15.0			13.8	14.1		
F-statistic			1.3	1.3			2.8	2.6
p = a	.237	.132	.248	.259	.130	.170	.009	.013

Family planning decisions	.285	.261	-.224	-.232	-.229	-.222	-.169*	-.170*
	(.252)	(.251)	(.226)	(.222)	(.231)	(.233)	(.120)	(.126)
Economic status	-.082	-.060	.010	.028	-.034	-.036	.036	.037
	(.100)	(.097)	(.058)	(.058)	(.071)	(.073)	(.053)	(.053)
Family planning*economic		-.201		-.203†		.040		-.007
		(.185)		(.115)		(.163)		(.100)
Wald χ^2^	12.1	12.5			13.8	13.9		
F-statistic			.9	1.3			3.3	3.0
p = a	.207	.253	.501	.280	.129	.177	.003	.004

Aggregate empowerment	.320	.234	.067	.057	-.552*	-.589	-.173	.220
	(.396)	(.382)	(.234)	(.228)	(.285)	(.280)	(.214)	(.270)
Economic status	-.082	-.033	.001	.008	-.042	.074	.032	.032
	(.101)	(.104)	(.059)	(.063)	(.072)	(.072)	(.052)	(.052)
Aggregate*economic		-.473*		-.077		-.253		.005
		(.218)		(.192)		(.214)		(.202)
Wald χ^2^	11.8	17.9			15.9	17.7		
F-statistic			.9	.8			2.9	2.7
p = a	.228	.057	.550	.597	.070	.060	.007	.010

*p < 0.05 †p < 0.10.

Note: Models control for child gender and age; maternal age, height (wasting only), and ever attended school; household size; household head ever attended school; and polygyny.

a For χ2 or F; 9 degrees of freedom for Model 1, 10 for Model 2.

Among children 3–5 months old, Model 2 revealed that the association between purchasing decisions and wasting differed significantly by economic status, such that the prevalence of wasting was lower when mothers simultaneously had more input into purchasing decisions and lived in economically better-off households (β_purchasing_ = 0.426, ns; β_economic_ = −0.038, ns; β_interaction_ = −0.642 p < 0.05). Similarly, a significant interaction was found for healthcare decisions: 3-5-month-olds were less likely to be wasted if they lived in households that were both economically better-off and mothers had more input into healthcare decisions, though the interaction was only marginally significant (β_healthcare_ = 0.330, ns; β_economic_ = -0.060, ns; β_interaction_ = −0.330 p < 0.10). This same pattern was significant for the aggregate empowerment score (β_aggregate_ = 0.234, ns; β_economic_ = −0.033, ns; β_interaction_ = −0.475, p < 0.05). There were no significant interactions for wasting among 6-12-month-olds or Hb among either age group.

### 3.3 Mediation analysis

The model predicting the change in wasting, which tested the simultaneous indirect effect of change in purchasing decisions, spousal communication, healthcare decision, and family planning decisions, fit well according to the CFI (0.952), TLI (0.945), and RMSEA (0.036) (Table 4, columns with β and CIs under Δwasting). The *a* paths, which describe the impact of the program on each domain of empowerment, revealed that the E-HFP program significantly increased purchasing decisions (β = 0.093; CI=(0.044, 0.145)) and spousal communication (β = 0.149; CI=(0.084, 0.214)). However, it did not significantly impact healthcare decisions (β = 0.042; CI=(-0.016, 0.100)) or family planning decisions (β = 0.047; CI=(-0.009, 0.113)). According to the LCSs, values at T_1_ were significantly and inversely associated with increases in empowerment, indicating that the largest increases in empowerment occurred among the least empowered at T_1_.

**Table 4:** Results of structural equation models that simultaneously test four domains of women's empowerment as mediators between the E-HFP program and changes in wasting and hemoglobin concentration and summary of direct, indirect, and total effects calculated from these models.

Outcome (Δnutritional status) =	Δ Wasting			ΔHemoglobin (g/dL)		
n =	1035			1035		
	β	LCI	UCI	β	LCI	UCI
**Select path coefficients from the structural portion of the structural equation model results**^a^						
**ΔPurchasing decisions** (equation 1)						
E-HFP (a_pur_ path)	.093*	.044	.145	.093*	.041	.145
Purchasing decisions T_1_ (LCS)	-.113*	-.128	-.100	-.113*	-.128	-.099
**ΔSpousal communication** (equation 2)						
E-HFP (a_com_ path)	.149*	.084	.214	.149*	.086	.214
Spousal communication T_1_ (LCS)	-.130*	-.162	-.103	-.130*	-.164	-.103
**ΔHealthcare decisions** (equation 3)						
E-HFP (a_hlth_ path)	.042	-.016	.100	.041	-.017	.099
Healthcare decisions T_1_ (LCS)	-.053*	-.072	-.036	-.054*	-.073	-.036
**ΔFamily planning decisions** (equation 4)						
E-HFP (a_fp_ path)	.047	-.009	.113	.047	-.017	.111
Family planning decisions T_1_ (LCS)	-.079*	-.108	-.055	-.078*	-.109	-.051
**ΔNutritional status** (equation 5)						
E-HFP (*c*’ path)	-.056	-.125	.016	.292*	.007	.561
ΔPurchasing decisions (b_pur_ path)	-.031	-.109	.050	-.147	-.452	.163
ΔSpousal communication (b_com_ path)	-.065*	-.128	-.004	.108	-.134	.358
ΔHealthcare decisions (b_hlth_ path)	-.073	-.216	.061	-.313	-.911	.243
ΔFamily planning decisions (b_fp_ path)	.045	-.072	.176	.064	-.473	.744
						
**Fit statistics**						
CFI	.954			.954		
TLI	.947			.947		
RMSEA	.035			.036		
**Direct, indirect, and total effects calculated from the path coefficients in the structural equation models**						
**Direct effect**						
of E-HFP (*c*’ path)	-.056	-.125	.016	.292*	.007	.561
**Individual indirect effects**^b^						
via Δpurchasing decisions (a_pur_*b_pur_)	-.003	-.011	.005	-.014	-.049	.016
via Δspousal communication (a_com_*b_com_)	-.010*	-.022	-.001	.016	-.020	.058
via Δhealthcare decisions (a_hlth_*b_hlth_)	-.003	-.014	.003	-.013	-.054	.017
via Δfamily planning decisions (a_fp_*b_fp_)	-.003	-.015	.003	-.015	-.061	.017
**Total indirect effect**^c^						
via Δ4 empowerment domains (Σ a*b)	-.019*	-.045	-.002	-.025	-.119	.052
**Total effect**^d^						
of E-HFP (c path)	-.075*	-.146	-.003	.266†	-.004	.535

*95% CI significant, †90% CI significant.

Note: All coefficients are unstandardized and correspond to the paths depicted in Fig. 1. Fixed parameters, covariates, observed items for latent variables, additional covariance terms, variance terms, and 90% confidence intervals are not shown here, and are available in Supplementary Tables 4 and 5. E-HFP = Enhanced-Homestead Food Production, LCI = lower confidence interval (95%), UCI = upper confidence interval (95%), LCS = Latent change score, CFI=Comparative Fit Index, TLI = Tucker-Lewis Index, RMSEA = Root Mean Square Error of Approximation.

a Variables shown in bold are the left-hand side variables for each equation. Variables that are indented and below each left-hand side variable are the right-hand side variables for each equation.

b Indirect effects for each mediator are calculated from the respective *a* and *b* paths using bootstrapping.

c Calculated as the sum of the four indirect effects of the four domains of women's empowerment.

d Calculated as the sum of the direct effect and the total indirect effect.

The *b* paths, which are the associations between each mediator and the change in wasting, revealed that increased spousal communication was significantly associated with a decrease in wasting (β = −0.065; CI=(-0.128, -0.004)). The changes in purchasing and healthcare decisions were both associated with change in wasting in the expected direction, but neither were significant. The association between the change in family planning decisions and the change in wasting was neither significant, nor in the expected direction.

Regarding the indirect effects via change in women's empowerment, we find that changes in purchasing, healthcare, and family planning decisions all contributed small amounts (-0.003) to the reduction in wasting. The independent contribution of change in spousal communication was the largest of the four and significant (β = −0.010; CI=(-0.022, -0.001)). The combined indirect effect of change in the four domains was also significant (β = −0.019; CI=(-0.045, -0.002)). The sum of the indirect and direct (total) effects revealed that the E-HFP program significantly reduced wasting (β = −0.075; CI=(-0.146, -0.003)). The ratio of the indirect effect to total effects suggests that 1.9 of the overall 7.5 percentage point reduction in wasting was attributable to changes in empowerment. In addition to these primary substantive results presented in Table 4, Supplementary Online Table 4 presents the complete results of this model, which include the measurement component, the associations between the baseline endogenous variables, the correlated error terms that were included, and the variances and means of all observed variables.

The model that predicted the change in Hb also fit well according to the CFI (0.954), TLI (0.947), and RMSEA (0.036) (Table 4, columns with β and CIs under Δhemoglobin). The *a* paths in the model for Hb, are nearly identical to the *a* paths in the wasting model, as are the associations between each domain of empowerment at T_1_ and its change score. The b paths, which were expected to be positive, reveal no significant associations between the change in Hb and the change in any of the empowerment domains, nor are the patterns suggestive of such. Turning to the indirect effects, we find that they did not account for the increase in Hb. The combined indirect effects of change in the four domains of women's empowerment was also not significant. Overall, the program had a moderately significant impact on Hb (β = 0.266; CI=(-0.004, 0.535)), which is similar to the impacts estimated for the full sample (Olney et al., 2015). Supplementary Table 5 presents the complete results of this model.

We also fit models with a single domain of empowerment at a time. There were minimal differences between the coefficients and indirect effects estimated for the models with a single domain and those for that same domain in the models that included all four domains. Thus, we do not report these results.

## 4 Discussion

Our study partially confirmed the hypotheses that women's empowerment is associated with a lower likelihood of child wasting and that household economic status moderates the association between women's empowerment and child wasting. We found evidence of a marginally significant main effect linking a lower prevalence of wasting to purchasing decisions and aggregate empowerment among 6-12-month-olds, but no evidence that this effect was moderated by household economic status. Among 3-5-month-olds, we found evidence that mothers who were simultaneously empowered—with respect to purchasing decisions, healthcare decisions (marginally significant), and aggregate empowerment—and lived in households with higher economic status had children with a lower prevalence of child wasting (a moderating effect). This conditional relationship suggests the importance of empowerment as a tool by which women leverage available resources in favor of their children's health. These findings should be interpreted in the context of Burkina Faso, a low-income country where most households lack basic resources and childhood undernutrition is prominent. It is possible that different conclusions would be reached in a better-off context. Moreover, we found no evidence in either age-group that women's empowerment was associated with higher Hb.

This study also found evidence that empowering women is a plausible pathway by which the E-HFP program, a nutrition- and gender-sensitive agricultural program, reduced the prevalence of wasting in the study population. We estimated that increases in women's empowerment accounted for 1.9 of the overall 7.5 percentage points reduction in wasting. This suggests that mothers who were empowered by the E-HFP program were able to translate program inputs (e.g., agricultural inputs and training and BCC programming) into improved WHZ for their children. Though a range of findings have suggested a cross-sectional association between empowerment and child nutrition (Cunningham et al., 2014; Hindin, 2000; van den Bold et al., 2013), this is the first empirical evidence implicating women's empowerment as a plausible program pathway in a nutrition-sensitive program. This pattern, however, was only the case for wasting; we found no evidence that women's empowerment mediated the increase in children's Hb.

The cross-sectional and mediation analyses both reach conclusions that are consistent with Kabeer's (2005) theory that empowering women can have instrumental value when it enables the use of resources. In this case, women's empowerment, whether in conjunction with household economic status or a nutrition-sensitive agricultural program, is linked to lower levels of child wasting. Taken together, these findings lend evidence to the broader theory that empowering women operates, in part, through enabling women to take advantage of resources. These findings have programmatic implications. They emphasize the importance of including women's empowerment as an explicit component of programs that aim to reduce child wasting (and possibly other nutrition outcomes), but in resource-constrained environments, programs also need to provide the minimum level of resources necessary to enable women to adopt practices that promote better child nutrition and health outcomes. Though the results are specific to an integrated agriculture, nutrition, and health program, it is possible that evidence linking women's empowerment and child nutritional status will be found in similar work on other nutrition-sensitive programs. More recently designed gender- and nutrition-sensitive agriculture programs have included stronger gender programming components. Such programs have the potential to drive even larger impacts on nutritional outcomes vis-à-vis women's empowerment. Future evaluations of nutrition-sensitive programs aimed at reducing poverty, food insecurity and undernutrition should consider testing women's empowerment as a pathway of impact.

One question raised by these findings is why women's empowerment is not associated with higher Hb in children in either the cross-sectional or longitudinal mediation analysis. The lack of significant findings and occasional findings in the opposite direction (i.e., association with spousal communication and family planning decisions among 6-12-month-olds in the cross-sectional models) were surprising. We found no previous studies that specifically examined the association between women's empowerment and child anemia or Hb. However, children in India whose mothers experienced domestic violence were more likely to be undernourished according to anthropometric indicators, but did not differ on their anemia status (Ackerson and Subramanian, 2008). One explanation for these findings may be that mothers have limited knowledge of anemia's multiple determinants, which prevents them from taking actions to reduce it (e.g., providing iron-rich foods or supplements, using bednets or other means of malaria prevention, and treating malaria). An additional explanation for these findings may be that the more obvious signs of low Hb in children (fatigue, loss of energy) may not be recognized in a context where most children are anemic. On the other hand, when signs of illness and energy deprivation are present, mothers may more easily identify them and potentially engage the support of others to these concerns.

Additionally, we found different patterns between 3-5-month-olds and 6-12-month-olds in which household economic status moderated the relationship between empowerment and wasting among the younger age group, but not the older one. One explanation is that mothers are most restricted in their physical mobility when their children are younger. During this time, they may be unable to generate income or cultivate food for themselves. Instead, they must rely heavily on other members of the household and their ability to gain access to existing household resources.

Finally, the findings also raise questions as to why different domains of empowerment were significant in the cross-sectional analysis and others in the mediation analysis. The cross-sectional analyses reinforce the importance of economic and healthcare decision making, which is consistent with a number of earlier findings (Malapit and Quisumbing, 2015; Somé et al., 2013). In the mediation analysis, however, spousal communication was responsible for the largest share of the indirect effect of the E-HFP program on wasting. The program's impact on spousal communication and purchasing decisions was larger (and significant) compared to healthcare and family planning decisions, and the association between the increase in spousal communication and the decrease in wasting was also strong. The program's BCC strategy encouraged mothers to communicate with their husbands and other household members to promote the adoption of the nutrition-specific actions taught by the program, and an evaluation of how the program was implemented found that mothers were seeking and receiving support from family members in adopting these actions (Olney, Behrman, Iruhiriye, van den Bold and Pedehombga, 2013). Additionally, related evidence found that the E-HFP program also improved men's perceptions of women as farm managers, suggesting increased respect and communication around agri-business activities (van den Bold et al., 2015), which may have bolstered women's success as farmers and earned them respect in other aspects of community life. On the other hand, family planning was not part of the BCC curriculum, and the intervention did not aim to expand the limited availability of family planning or healthcare services. These factors may have limited the potential for women to be empowered in these domains. Furthermore, it may be the case that only certain domains of women's empowerment are instrumental for improving child nutritional status, and these may depend on the context. For example, spousal communication may be particularly important in contexts where men hold most of the financial resources and can exercise veto power over healthcare seeking. Other domains of women's empowerment, however, may still be important for their instrumental value for improving other outcomes or their intrinsic value.

### 4.1 Limitations

First, the mediation analysis is limited by the frequency of measurement; only baseline and follow-up data from a two-year-long program were available. A more comprehensive understanding of how these processes unfold, especially the temporal ordering of women's empowerment and nutritional outcomes could be achieved with more frequent data collection (Preacher, 2015).

The study is also limited by the number of items available to assess women's empowerment in the domains of healthcare and family planning decisions. Only two items were used for these two latent constructs, and their underlying measurement structure was relatively weak. Additionally, questions related to IYCF could not be included. The data were collected before the Women's Empowerment in Agriculture Index was developed (Alkire et al., 2013). Future related studies would benefit from empowerment metrics that have undergone rigorous psychometric evaluation and address additional aspects of women's empowerment, such as intrinsic agency, mobility, political representations, and collective agency (Ewerling et al., 2017; Sundström et al., 2017; Yount et al., 2018).

## 5 Conclusion

Overall, this study contributes to the literature linking women's empowerment to child anthropometric status (specifically wasting). By examining women's empowerment as a mediating factor in the impact of a nutrition-sensitive agricultural program, this study provides the first experimental evidence that empowering women is a pathway to achieve impacts on child nutritional status. Thus, empowering women provides dual benefits: it improves women's lives and in turn, conveys tangible benefits for their children's nutrition. Women's empowerment may also be a pathway to reduce child wasting in other types of nutrition-sensitive programs, and may improve other nutritional status indicators (including HAZ or stunting) in programs that are implemented and evaluated over longer periods of time. Future evaluations of gender- and nutrition-sensitive programs should test these pathways so that programs can be better designed to foster both women's empowerment and child nutrition.
