# Supplementary material for: Is women's empowerment a pathway to improving child nutrition outcomes in a nutrition-sensitive agriculture program?: Evidence from a randomized controlled trial in Burkina Faso
Source: Soc Sci Med. 2019 Jul;233:93–102. doi: 10.1016/j.socscimed.2019.05.016 (PMC6642337; doi:10.1016/j.socscimed.2019.05.016)
Supplement: Supplementary tables [file mmc1.docx]

**Supplementary Online Content**

**Supplementary Table 1: Pattern matrix results of exploratory factor analysis**

| **Variable** | **Factor 1** | **Factor 2** | **Factor 3** |
| --- | --- | --- | --- |
| **Decisions to purchase** |  |  | -- |
| Small quantities of food | -- | .67 | -- |
| Large quantities of food | -- | .75 | -- |
| Clothing for self | -- | .75 | -- |
| Medicine for self | -- | .68 | -- |
| Toiletries | -- | .69 | -- |
| Medication for children | -- | .72 | -- |
| Special food for children | -- | .72 | -- |
| **Talks to spouse about** |  |  |  |
| Professional and agricultural activities | .50 | -- | -- |
| Domestic activities | .61 | -- | -- |
| Expenses | .79 | -- | -- |
| Community events | .85 | -- | -- |
| Child's health | .76 | -- | -- |
| Child's nutrition | .82 | -- | -- |
| Personal health | .71 | -- | -- |
| **Healthcare decisions** |  |  |  |
| When child is sick | -- | -- | .43 |
| Doctor when pregnant | -- | -- | .44 |
| **Family planning decisions** |  |  |  |
| Use contraception | -- | -- | .51 |
| Have another child | -- | -- | 50 |

--< |.3|

Note: Results are orthogonal (varimax) rotation. Factor 3 was divided into two factors (healthcare decisions and family planning decisions), based on theoretical justification.

**Supplementary Table 2: Results of confirmatory factor analysis models for four women’s empowerment factors at baseline and follow-up**

|  |  | Baseline (T1) | | Follow-up (T_2_) | |
| --- | --- | --- | --- | --- | --- |
| Latent construct | Manifest Variable | Factor loading | t value | Factor loading | t value |
| Purchasing decisions | Contributes to decisions to purchase... |  |  |  |  |
|  | Small quantities of food | .50 | 20.02*** | .55 | 23.35*** |
|  | Large quantities of food | .67 | 31.61*** | .59 | 27.47*** |
|  | Clothing for self | .64 | 29.16*** | .85 | 62.71*** |
|  | Medicine for self | .95 | 48.23*** | .77 | 36.78*** |
|  | Toiletries | .63 | 31.32*** | .74 | 41.13*** |
|  | Medication for children | .85 | 45.65*** | .94 | 81.82*** |
|  | Special food for children | .77 | 36.49*** | .75 | 34.01*** |
| Spousal communication | Talks to spouse about… |  |  |  |  |
|  | Professional and agricultural activities | .73 | 33.28*** | .87 | 88.37*** |
|  | Domestic activities | .83 | 56.48*** | .91 | 129.78*** |
|  | Expenses | .85 | 57.99*** | .94 | 148.17*** |
|  | Community events | .69 | 35.77*** | .81 | 69.19*** |
|  | Child's health | .40 | 13.52*** | .74 | 50.77*** |
|  | Child's nutrition | .47 | 17.87*** | .78 | 59.63*** |
|  | Personal health | .41 | 14.88*** | .77 | 56.42*** |
| Healthcare decisions | Contributes to decisions about… |  |  |  |  |
|  | When child is sick | .67 | 23.22*** | .79 | 39.11*** |
|  | Doctor when pregnant | .62 | 21.51*** | .78 | 38.43*** |
| Family planning decisions | Contributes to decisions about… |  |  |  |  |
|  | Use contraception | .79 | 27.50*** | .90 | 27.51*** |
|  | Have another child | .62 | 21.97*** | .52 | 17.63*** |
| Fit statistics |  |  |  |  |  |
| CFI |  | .968 |  | .975 |  |
| TLI |  | .958 |  | .967 |  |
| RMSEA |  | .048 |  | .055 |  |

***p<.001

Note: n=1035 for both time points. CFI=Comparative Fit Index, TLI=Tucker-Lewis Index, RMSEA=Root Mean Square Error of Approximation

**Supplementary Table 3:** Descriptive characteristics and correlation table of individual items (manifest variables) used to construct latent variables of women’s empowerment

|  |  | Sample means | | |  |  |  |  |  |  |  |  |
| --- | --- | --- | --- | --- | --- | --- | --- | --- | --- | --- | --- | --- |
|  |  | Total | Tx Arm | Ctrl  Arm | 1 | 2 | 3 | 4 | 5 | 6 | 7 | 8 |
|  | **Baseline (T_1_)** |  |  |  |  |  |  |  |  |  |  |  |
|  | **Decisions to purchase** |  |  |  |  |  |  |  |  |  |  |  |
| 1 | Small quantities of food | .82 | .79 | .85 | 1.00 |  |  |  |  |  |  |  |
| 2 | Large quantities of food | .37 | .34 | .41 | .36 | 1.00 |  |  |  |  |  |  |
| 3 | Clothing for self | .71 | .73 | .69 | .52 | .43 | 1.00 |  |  |  |  |  |
| 4 | Medicine for self | .60 | .57 | .65 | .47 | .53 | .71 | 1.00 |  |  |  |  |
| 5 | Toiletries | .74 | .74 | .76 | .32 | .40 | .64 | .61 | 1.00 |  |  |  |
| 6 | Medication for children | .50 | .48 | .57 | .37 | .57 | .54 | .75 | .52 | 1.00 |  |  |
| 7 | Special food for children | .58 | .57 | .60 | .40 | .52 | .47 | .54 | .47 | .73 | 1.00 |  |
|  | **Talks to spouse about** |  |  |  |  |  |  |  |  |  |  |  |
| 8 | Professional and agricultural activities | 1.54 | 1.55 | 1.52 | .11 | .16 | .10 | .12 | .11 | .11 | .16 | 1.00 |
| 9 | Domestic activities | 1.48 | 1.49 | 1.46 | .08 | .18 | .08 | .12 | .09 | .11 | .12 | .61 |
| 10 | Expenses | 1.45 | 1.47 | 1.42 | .02 | .14 | .01 | .07 | .03 | .06 | .04 | .54 |
| 11 | Community events | 1.29 | 1.28 | 1.31 | .15 | .22 | .14 | .19 | .10 | .15 | .14 | .58 |
| 12 | Child's health | 1.75 | 1.79 | 1.68 | -.06 | .11 | .02 | .07 | .07 | .05 | .10 | .22 |
| 13 | Child's nutrition | 1.69 | 1.71 | 1.67 | -.05 | .09 | .02 | .08 | .08 | .03 | -.01 | .32 |
| 14 | Personal health | 1.74 | 1.78 | 1.69 | -.06 | .09 | .01 | .05 | .09 | .01 | .04 | .28 |
|  | **Health decisions about** |  |  |  |  |  |  |  |  |  |  |  |
| 15 | When child is sick | .60 | .58 | .63 | .14 | .17 | .14 | .19 | .08 | .26 | .30 | .11 |
| 16 | Doctor when pregnant | .65 | .58 | .75 | .20 | .22 | .18 | .30 | .11 | .31 | .25 | .14 |
|  | **Family planning decisions** |  |  |  |  |  |  |  |  |  |  |  |
| 17 | Use contraception | .64 | .62 | .67 | .23 | .17 | .24 | .27 | .17 | .24 | .24 | .11 |
| 18 | Have another child | .52 | .51 | .52 | .22 | .19 | .20 | .24 | .13 | .25 | .26 | .12 |
|  | **Follow-up (T_2_)** |  |  |  |  |  |  |  |  |  |  |  |
|  | **Decisions to purchase** |  |  |  |  |  |  |  |  |  |  |  |
| 19 | Small quantities of food | .84 | .85 | .83 | .10 | .01 | .09 | .09 | .02 | .05 | .07 | -.06 |
| 20 | Large quantities of food | .42 | .45 | .38 | .07 | .07 | .05 | .07 | .05 | .08 | .08 | .03 |
| 21 | Clothing for self | .71 | .76 | .66 | .06 | .02 | .04 | .06 | .03 | .03 | .06 | .01 |
| 22 | Medicine for self | .50 | .53 | .45 | .05 | .02 | .02 | .02 | .02 | .02 | .07 | .04 |
| 23 | Toiletries | .79 | .82 | .76 | .12 | .03 | .09 | .10 | .07 | .07 | .12 | .03 |
| 24 | Medication for children | .69 | .72 | .65 | .08 | .02 | .06 | .06 | .06 | .03 | .08 | .01 |
| 25 | Special food for children | .49 | .51 | .46 | .03 | .03 | .02 | .03 | .04 | .03 | .05 | .05 |
|  | **Talks to spouse about** |  |  |  |  |  |  |  |  |  |  |  |
| 26 | Professional and agricultural activities | 1.40 | 1.46 | 1.32 | .10 | .02 | .08 | .04 | .09 | .04 | .04 | .02 |
| 27 | Domestic activities | 1.38 | 1.42 | 1.31 | .12 | .02 | .11 | .06 | .11 | .03 | .03 | .01 |
| 28 | Expenses | 1.40 | 1.46 | 1.33 | .13 | .02 | .10 | .05 | .11 | .05 | .04 | -.01 |
| 29 | Community events | 1.31 | 1.35 | 1.25 | .14 | .04 | .09 | .03 | .11 | .06 | .08 | .02 |
| 30 | Child's health | 1.53 | 1.59 | 1.43 | .07 | .02 | .05 | .01 | .04 | .04 | .05 | -.05 |
| 31 | Child's nutrition | 1.49 | 1.55 | 1.41 | .09 | .02 | .05 | .02 | .04 | .06 | .08 | -.05 |
| 32 | Personal health | 1.51 | 1.58 | 1.42 | .08 | .02 | .05 | .02 | .05 | .05 | .05 | -.05 |
|  | **Health decisions about** |  |  |  |  |  |  |  |  |  |  |  |
| 33 | When child is sick | .50 | .52 | .48 | .01 | .00 | -.01 | .03 | .00 | .03 | .05 | .04 |
| 34 | Doctor when pregnant | .50 | .50 | .50 | .02 | .04 | .02 | .06 | .03 | .07 | .12 | .05 |
|  | **Family planning decisions** |  |  |  |  |  |  |  |  |  |  |  |
| 35 | Use contraception | .46 | .47 | .46 | .10 | .03 | .07 | .08 | .05 | .07 | .09 | -.01 |
| 36 | Have another child | .41 | .42 | .41 | .05 | .02 | .07 | .08 | .06 | .07 | .08 | .01 |

|  | 9 | 10 | 11 | 12 | 13 | 14 | 15 | 16 | 17 | 18 | 19 | 20 | 21 | 22 | 23 | 24 |
| --- | --- | --- | --- | --- | --- | --- | --- | --- | --- | --- | --- | --- | --- | --- | --- | --- |
| 9 | 1.00 |  |  |  |  |  |  |  |  |  |  |  |  |  |  |  |
| 10 | .70 | 1.00 |  |  |  |  |  |  |  |  |  |  |  |  |  |  |
| 11 | .57 | .59 | 1.00 |  |  |  |  |  |  |  |  |  |  |  |  |  |
| 12 | .34 | .35 | .28 | 1.00 |  |  |  |  |  |  |  |  |  |  |  |  |
| 13 | .38 | .43 | .31 | .64 | 1.00 |  |  |  |  |  |  |  |  |  |  |  |
| 14 | .33 | .37 | .32 | .72 | .67 | 1.00 |  |  |  |  |  |  |  |  |  |  |
| 15 | .09 | .06 | .13 | .06 | .05 | .00 | 1.00 |  |  |  |  |  |  |  |  |  |
| 16 | .09 | .10 | .20 | -.02 | .03 | -.04 | .42 | 1.00 |  |  |  |  |  |  |  |  |
| 17 | .05 | .08 | .11 | -.01 | .03 | -.04 | .39 | .42 | 1.00 |  |  |  |  |  |  |  |
| 18 | .15 | .11 | .10 | -.08 | -.04 | -.08 | .32 | .28 | .49 | 1.00 |  |  |  |  |  |  |
| 19 | -.03 | -.03 | -.05 | .02 | .01 | .03 | .06 | .00 | .10 | .09 | 1.00 |  |  |  |  |  |
| 20 | -.01 | -.02 | .03 | .05 | -.03 | .01 | .06 | -.01 | .04 | .05 | .31 | 1.00 |  |  |  |  |
| 21 | -.02 | -.02 | -.02 | .06 | .03 | .04 | .06 | .00 | .09 | .10 | .54 | .49 | 1.00 |  |  |  |
| 22 | -.01 | -.04 | -.04 | .05 | .01 | .01 | .05 | -.02 | .05 | .05 | .36 | .58 | .62 | 1.00 |  |  |
| 23 | -.01 | -.03 | -.04 | .01 | -.01 | .02 | .12 | .04 | .13 | .11 | .63 | .41 | .73 | .49 | 1.00 |  |
| 24 | -.03 | -.03 | -.03 | .05 | .01 | .04 | .09 | .03 | .10 | .08 | .54 | .51 | .81 | .61 | .72 | 1.00 |
| 25 | -.02 | -.02 | -.03 | .04 | -.01 | .01 | .06 | -.01 | .07 | .04 | .36 | .59 | .57 | .85 | .48 | .65 |
| 26 | .02 | .07 | .03 | .03 | .02 | .06 | .06 | .04 | .08 | .07 | .12 | .19 | .13 | .06 | .19 | .16 |
| 27 | .01 | .03 | -.02 | -.01 | -.01 | .01 | .07 | .05 | .08 | .05 | .10 | .16 | .08 | .05 | .17 | .13 |
| 28 | .02 | .07 | .03 | -.01 | .00 | .03 | .08 | .05 | .09 | .08 | .11 | .17 | .10 | .03 | .18 | .15 |
| 29 | .02 | .06 | .01 | .00 | -.02 | .02 | .05 | .04 | .07 | .07 | .07 | .16 | .08 | .04 | .15 | .13 |
| 30 | -.01 | .06 | -.01 | .01 | .01 | .03 | .05 | .06 | .09 | .08 | .09 | .16 | .11 | .07 | .18 | .12 |
| 31 | -.03 | .04 | -.02 | -.01 | -.01 | .02 | .09 | .09 | .14 | .12 | .04 | .13 | .07 | .01 | .16 | .08 |
| 32 | -.01 | .05 | -.02 | .01 | .01 | .04 | .06 | .06 | .12 | .10 | .06 | .16 | .10 | .04 | .16 | .11 |
| 33 | -.02 | -.06 | -.03 | .01 | -.04 | -.02 | .05 | .02 | .06 | .03 | .18 | .20 | .25 | .37 | .27 | .25 |
| 34 | .04 | .00 | -.07 | .05 | -.03 | -.01 | .10 | -.02 | .08 | .10 | .16 | .18 | .23 | .34 | .28 | .22 |
| 35 | -.01 | -.03 | -.05 | -.03 | -.02 | -.02 | .14 | .04 | .17 | .16 | .15 | .14 | .19 | .16 | .23 | .18 |
| 36 | -.02 | -.03 | .00 | -.02 | -.02 | -.02 | .10 | .02 | .17 | .16 | .08 | .19 | .09 | .15 | .14 | .12 |
|  | 25 | 26 | 27 | 28 | 29 | 30 | 31 | 32 | 33 | 34 | 35 | 36 |  |  |  |  |
| 25 | 1.00 |  |  |  |  |  |  |  |  |  |  |  |  |  |  |  |
| 26 | .08 | 1.00 |  |  |  |  |  |  |  |  |  |  |  |  |  |  |
| 27 | .05 | .80 | 1.00 |  |  |  |  |  |  |  |  |  |  |  |  |  |
| 28 | .05 | .76 | .85 | 1.00 |  |  |  |  |  |  |  |  |  |  |  |  |
| 29 | .04 | .69 | .75 | .76 | 1.00 |  |  |  |  |  |  |  |  |  |  |  |
| 30 | .04 | .64 | .65 | .72 | .59 | 1.00 |  |  |  |  |  |  |  |  |  |  |
| 31 | .02 | .66 | .70 | .75 | .62 | .86 | 1.00 |  |  |  |  |  |  |  |  |  |
| 32 | .04 | .66 | .68 | .73 | .62 | .91 | .89 | 1.00 |  |  |  |  |  |  |  |  |
| 33 | .38 | -.02 | -.01 | -.05 | .00 | .01 | -.04 | -.01 | 1.00 |  |  |  |  |  |  |  |
| 34 | .34 | -.02 | -.03 | -.06 | -.02 | .04 | -.02 | .02 | .61 | 1.00 |  |  |  |  |  |  |
| 35 | .17 | .10 | .10 | .08 | .08 | .10 | .08 | .12 | .48 | .51 | 1.00 |  |  |  |  |  |
| 36 | .17 | .10 | .09 | .09 | .07 | .10 | .12 | .14 | .30 | .25 | .47 | 1.00 |  |  |  |  |

**Supplementary Table 4**: Full results of a structural equation model that simultaneously tests four domains of women’s empowerment as mediators between the E-HFP program and changes in child wasting and summary of direct, indirect, and total effects calculated from these models.

|  |  | β | LCI 95% | UCI 95% | LCI 90% | UCI 90% |
| --- | --- | --- | --- | --- | --- | --- |
| **Structural equation model result^1^** |  |  |  |  |  |  |
| **Structural model** |  |  |  |  |  |  |
| **(left-hand side variable)** | **(right-hand side variables)** |  |  |  |  |  |
| ∆Purchasing decisions (equation 1) | E-HFP (a_pur_ path) | .093 | .044 | .145 | .055 | .135 |
|  | Child sex (male=1) | -.021 | -.071 | .027 | -.063 | .019 |
|  | Child age (months) | .000 | -.008 | .008 | -.008 | .007 |
|  | Maternal age (years) | .001 | -.003 | .005 | -.002 | .004 |
|  | Maternal height (cm) | .001 | -.003 | .006 | -.002 | .005 |
|  | Household economic status | -.012 | -.031 | .008 | -.028 | .005 |
|  | Maternal education (any 0/1) | -.052 | -.160 | .058 | -.142 | .040 |
|  | Household head education (any 0/1) | .015 | -.073 | .109 | -.057 | .091 |
| ∆Spousal communication (equation 2) | E-HFP (a_com_ path) | .149 | .084 | .214 | .096 | .205 |
|  | Child sex (male=1) | -.029 | -.092 | .028 | -.083 | .020 |
|  | Child age (months) | -.009 | -.020 | .004 | -.017 | .001 |
|  | Maternal age (years) | .005 | .000 | .010 | .001 | .009 |
|  | Maternal height (cm) | .004 | -.001 | .009 | .000 | .008 |
|  | household economic status | .016 | -.011 | .043 | -.005 | .039 |
|  | Maternal education (any 0/1) | -.032 | -.167 | .108 | -.149 | .082 |
|  | Household head education (any 0/1) | -.031 | -.139 | .085 | -.121 | .070 |
| ∆Healthcare decisions (equation 3) | E-HFP (a_hlth_ path) | .042 | -.016 | .100 | -.005 | .090 |
|  | Child sex (male=1) | .049 | -.009 | .106 | .003 | .097 |
|  | Child age (months) | -.003 | -.013 | .007 | -.012 | .005 |
|  | Maternal age (years) | -.004 | -.009 | .000 | -.008 | .000 |
|  | Maternal height (cm) | .000 | -.004 | .005 | -.004 | .004 |
|  | Household economic status | -.003 | -.027 | .021 | -.023 | .017 |
|  | Maternal education (any 0/1) | .038 | -.081 | .152 | -.058 | .135 |
|  | Household head education (any 0/1) | -.021 | -.120 | .077 | -.103 | .064 |
| ∆Family planning decisions (equation 4) | E-HFP (a_fp_ path) | .047 | -.009 | .113 | -.001 | .097 |
|  | Child sex (male=1) | .030 | -.031 | .088 | -.019 | .079 |
|  | Child age (months) | .000 | -.012 | .011 | -.009 | .009 |
|  | Maternal age (years) | -.003 | -.009 | .002 | -.008 | .001 |
|  | Maternal height (cm) | .002 | -.003 | .006 | -.002 | .006 |
|  | Household economic status | -.002 | -.025 | .023 | -.022 | .019 |
|  | Maternal education (any 0/1) | -.010 | -.147 | .112 | -.124 | .093 |
|  | Household head education (any 0/1) | .024 | -.078 | .125 | -.062 | .108 |
| ∆Wasting (equation 5) | E-HFP (c' path) | -.056 | -.125 | .016 | -.114 | .004 |
|  | ∆Purchasing decisions (b_pur_ path) | -.031 | -.109 | .050 | -.094 | .038 |
|  | ∆Spousal communication (b_com_ path) | -.065 | -.128 | -.004 | -.118 | -.012 |
|  | ∆Healthcare decisions (b_hlth_ path) | -.073 | -.216 | .061 | -.191 | .043 |
|  | ∆Family planning decisions (b_fp_ path) | .045 | -.072 | .176 | -.053 | .150 |
|  | Child sex (male=1) | .042 | -.020 | .113 | -.010 | .101 |
|  | Child age (months) | -.016 | -.031 | -.004 | -.027 | -.006 |
|  | Maternal age (years) | -.003 | -.009 | .003 | -.008 | .002 |
|  | Maternal height (cm) | -.004 | -.010 | .002 | -.008 | .001 |
|  | Household economic status | .010 | -.094 | .110 | -.076 | .096 |
|  | Maternal education (any 0/1) | -.147 | -.761 | .499 | -.667 | .392 |
|  | Household head education (any 0/1) | -.234 | -.694 | .225 | -.606 | .147 |
| **Measurement components** |  |  |  |  |  |  |
| Purchasing decisions T1 | To buy medication for children T1 | 1.000 | 1.000 | 1.000 | 1.000 | 1.000 |
|  | To buy small amounts of food T1 | .607 | .556 | .658 | .563 | .649 |
|  | To buy large amounts of food T1 | .786 | .748 | .828 | .753 | .823 |
|  | To buy clothing for self T1 | 1.053 | 1.016 | 1.090 | 1.021 | 1.084 |
|  | To buy medicine for self T1 | 1.053 | 1.018 | 1.087 | 1.024 | 1.082 |
|  | To buy toiletries T1 | .883 | .842 | .929 | .849 | .922 |
|  | To buy foods for children T1 | .971 | .935 | 1.006 | .942 | 1.000 |
| Purchasing decisions T2 | To buy medication for children T2 | 1.000 | 1.000 | 1.000 | 1.000 | 1.000 |
|  | To buy small amounts of food T2 | .607 | .556 | .658 | .563 | .649 |
|  | To buy large amounts of food T2 | .786 | .748 | .828 | .753 | .823 |
|  | To buy clothing for self T2 | 1.053 | 1.016 | 1.090 | 1.021 | 1.084 |
|  | To buy medicine for self T2 | 1.053 | 1.018 | 1.087 | 1.024 | 1.082 |
|  | To buy toiletries T2 | .883 | .842 | .929 | .849 | .922 |
|  | To buy foods for children T2 | .971 | .935 | 1.006 | .942 | 1.000 |
| Spousal communication T1 | Talk about expenses T1 | 1.000 | 1.000 | 1.000 | 1.000 | 1.000 |
|  | Talk about prof/ag activities T1 | .916 | .869 | .963 | .876 | .953 |
|  | Talk about domestic activities T1 | .975 | .938 | 1.012 | .944 | 1.005 |
|  | Talk about community events T1 | .921 | .871 | .975 | .882 | .967 |
|  | Talk about child's health T1 | .735 | .671 | .795 | .682 | .787 |
|  | Talk about child nutrition T1 | .787 | .723 | .847 | .735 | .836 |
|  | Talk about own health T1 | .758 | .693 | .819 | .705 | .808 |
| Spousal communication T2 | Talk about expenses T2 | 1.000 | 1.000 | 1.000 | 1.000 | 1.000 |
|  | Talk about prof/ag activities T2 | .916 | .869 | .963 | .876 | .953 |
|  | Talk about domestic activities T2 | .975 | .938 | 1.012 | .944 | 1.005 |
|  | Talk about community events T2 | .921 | .871 | .975 | .882 | .967 |
|  | Talk about child's health T2 | .735 | .671 | .795 | .682 | .787 |
|  | Talk about child nutrition T2 | .787 | .723 | .847 | .735 | .836 |
|  | Talk about own health T2 | .758 | .693 | .819 | .705 | .808 |
| Healthcare decisions T1 | Decisions to seek care when pregnant T1 | 1.000 | 1.000 | 1.000 | 1.000 | 1.000 |
|  | Decisions for sick child T1 | .998 | .927 | 1.081 | .936 | 1.064 |
| Healthcare decisions T2 | Decisions to seek care when pregnant T2 | 1.000 | 1.000 | 1.000 | 1.000 | 1.000 |
|  | Decisions for sick child T2 | .998 | .927 | 1.081 | .936 | 1.064 |
| Family planning decisions T1 | Decisions to use contraception T1 | 1.000 | 1.000 | 1.000 | 1.000 | 1.000 |
|  | Decisions to have another child T1 | .678 | .600 | .750 | .611 | .740 |
| Family planning decisions T2 | Decisions to use contraception T2 | 1.000 | 1.000 | 1.000 | 1.000 | 1.000 |
|  | Decisions to have another child T2 | .678 | .600 | .750 | .611 | .740 |
| **Latent change score components** |  |  |  |  |  |  |
| **Latent change score for purchasing decisions** | |  |  |  |  |  |
| Purchasing decisions T2 | Purchasing decisions T1 | 1.000 | 1.000 | 1.000 | 1.000 | 1.000 |
| ∆Purchasing decisions | Purchasing decisions T2 | 1.000 | 1.000 | 1.000 | 1.000 | 1.000 |
| Purchasing decisions T1 |  | -.029 | -.114 | .055 | -.099 | .036 |
| ∆Purchasing decisions |  | -.204 | -.761 | .344 | -.643 | .237 |
| Purchasing decisions T1 | Purchasing decisions T1 | .132 | .122 | .143 | .124 | .141 |
| Purchasing decisions T1 | ∆Purchasing decisions | -.113 | -.128 | -.100 | -.125 | -.102 |
| ∆Purchasing decisions | ∆Purchasing decisions | .244 | .219 | .271 | .222 | .265 |
| **Latent change score for spousal communication** | |  |  |  |  |  |
| Spousal communication T2 | Spousal communication T1 | 1.000 | 1.000 | 1.000 | 1.000 | 1.000 |
| ∆Spousal communication | Spousal communication T2 | 1.000 | 1.000 | 1.000 | 1.000 | 1.000 |
| Spousal communication T1 |  | -.148 | -.314 | .025 | -.288 | -.001 |
| ∆Spousal communication |  | -.532 | -1.019 | -.023 | -.932 | -.103 |
| Spousal communication T1 | Spousal communication T1 | .132 | .107 | .163 | .110 | .158 |
| Spousal communication T1 | ∆Spousal communication | -.130 | -.162 | -.103 | -.156 | -.106 |
| ∆Spousal communication | ∆Spousal communication | .383 | .342 | .426 | .346 | .419 |
| **Latent change score for healthcare decisions** | |  |  |  |  |  |
| Healthcare decisions T2 | Healthcare decisions T1 | 1.000 | 1.000 | 1.000 | 1.000 | 1.000 |
| ∆Healthcare decisions | Healthcare decisions T2 | 1.000 | 1.000 | 1.000 | 1.000 | 1.000 |
| Healthcare decisions T1 |  | .017 | -.132 | .156 | -.100 | .133 |
| ∆Healthcare decisions |  | .003 | -.440 | .397 | -.363 | .353 |
| Healthcare decisions T1 | Healthcare decisions T1 | .087 | .071 | .102 | .075 | .099 |
| Healthcare decisions T1 | ∆Healthcare decisions | -.053 | -.072 | -.036 | -.067 | -.039 |
| ∆Healthcare decisions | ∆Healthcare decisions | .201 | .170 | .231 | .175 | .226 |
| **Latent change score for family planning decisions** | |  |  |  |  |  |
| Family planning decisions T2 | Family planning decisions T1 | 1.000 | 1.000 | 1.000 | 1.000 | 1.000 |
| ∆Family planning decisions | Family planning decisions T2 | 1.000 | 1.000 | 1.000 | 1.000 | 1.000 |
| Family planning decisions T1 |  | -.052 | -.194 | .105 | -.175 | .075 |
| ∆Family planning decisions |  | -.086 | -.442 | .289 | -.381 | .226 |
| Family planning decisions T1 | Family planning decisions T1 | .149 | .127 | .173 | .131 | .169 |
| Family planning decisions T1 | ∆Family planning decisions | -.079 | -.108 | -.055 | -.103 | -.058 |
| ∆Family planning decisions | ∆Family planning decisions | .228 | .184 | .275 | .190 | .269 |
| **Observed characteristics with correlated error terms** | |  |  |  |  |  |
| To buy small amounts of food T1 | To buy toiletries T1 | -.020 | -.028 | -.011 | -.026 | -.013 |
| To buy large amounts of food T1 | To buy clothing for self T1 | -.012 | -.019 | -.005 | -.018 | -.006 |
| To buy large amounts of food T1 | To buy medicine for self T1 | .014 | .004 | .024 | .006 | .022 |
| To buy large amounts of food T1 | To buy foods for children T1 | .032 | .020 | .044 | .022 | .041 |
| To buy clothing for self T1 | To buy foods for children T1 | -.018 | -.025 | -.011 | -.024 | -.012 |
| To buy medication for children T1 | To buy large amounts of food T1 | .047 | .035 | .059 | .037 | .057 |
| To buy medication for children T1 | To buy medicine for self T1 | .053 | .043 | .063 | .044 | .061 |
| To buy medication for children T1 | To buy toiletries T1 | .009 | .003 | .015 | .003 | .014 |
| To buy medication for children T1 | To buy foods for children T1 | .073 | .060 | .086 | .062 | .084 |
| To buy small amounts of food T2 | To buy toiletries T2 | .021 | .014 | .028 | .015 | .027 |
| To buy large amounts of food T2 | To buy foods for children T2 | .052 | .040 | .064 | .042 | .062 |
| To buy large amounts of food T2 | To buy medicine for self T2 | .048 | .037 | .060 | .038 | .058 |
| To buy clothing for self T2 | To buy foods for children T2 | -.006 | -.012 | .000 | -.011 | -.001 |
| To buy medicine for self T2 | To buy foods for children T2 | .099 | .087 | .112 | .089 | .110 |
| To buy medication for children T2 | To buy clothing for self T2 | .009 | .001 | .019 | .002 | .017 |
| To buy medication for children T2 | To buy foods for children T2 | .016 | .009 | .023 | .010 | .022 |
| Talk about expenses T1 | Talk about domestic activities T1 | .106 | .079 | .135 | .083 | .129 |
| Talk about prof/ag activities T1 | Talk about domestic activities T1 | .082 | .053 | .112 | .059 | .108 |
| Talk about child's health T1 | Talk about own health T1 | .092 | .075 | .112 | .077 | .108 |
| Talk about prof/ag activities T1 | Talk about community events T1 | .111 | .084 | .139 | .089 | .135 |
| Talk about child nutrition T1 | Talk about own health T1 | .088 | .068 | .108 | .072 | .105 |
| Talk about community events T1 | Talk about own health T1 | .013 | .000 | .025 | .002 | .024 |
| Talk about child's health T1 | Talk about child nutrition T1 | .079 | .061 | .097 | .064 | .094 |
| Talk about prof/ag activities T1 | Talk about child's health T1 | -.020 | -.033 | -.008 | -.030 | -.011 |
| Talk about expenses T1 | Talk about community events T1 | .104 | .074 | .132 | .079 | .127 |
| Talk about domestic activities T1 | Talk about community events T1 | .102 | .072 | .132 | .078 | .128 |
| Talk about expenses T1 | Talk about prof/ag activities T1 | .057 | .028 | .086 | .034 | .081 |
| Talk about expenses T1 | Talk about child nutrition T1 | .013 | .002 | .025 | .004 | .023 |
| Talk about expenses T2 | Talk about domestic activities T2 | -.010 | -.024 | .003 | -.022 | .002 |
| Talk about child's health T2 | Talk about own health T2 | .095 | .079 | .112 | .081 | .109 |
| Talk about prof/ag activities T2 | Talk about domestic activities T2 | .003 | -.012 | .018 | -.010 | .015 |
| Talk about child nutrition T2 | Talk about own health T2 | .083 | .067 | .101 | .069 | .098 |
| Talk about child's health T2 | Talk about child nutrition T2 | .080 | .063 | .098 | .066 | .094 |
| Talk about expenses T2 | Talk about prof/ag activities T2 | -.018 | -.028 | -.007 | -.027 | -.009 |
| **Correlations between mediators** |  |  |  |  |  |  |
| ∆Purchasing decisions | ∆Spousal communication | .015 | .001 | .028 | .003 | .026 |
| ∆Purchasing decisions | ∆Healthcare decisions | .052 | .040 | .064 | .042 | .061 |
| ∆Spousal communication | ∆Healthcare decisions | -.026 | -.040 | -.010 | -.038 | -.012 |
| ∆Purchasing decisions | ∆Family planning decisions | .027 | .013 | .039 | .015 | .037 |
| ∆Spousal communication | ∆Family planning decisions | .018 | .001 | .033 | .004 | .031 |
| ∆Healthcare decisions | ∆Family planning decisions | .100 | .082 | .115 | .085 | .112 |
| **Correlations among T1 variables (excluding measurement model)** | |  |  |  |  |  |
| Purchasing decisions T1 | Spousal communication T1 | .015 | .008 | .021 | .009 | .021 |
| Purchasing decisions T1 | Healthcare decisions T1 | .026 | .018 | .033 | .020 | .032 |
| Purchasing decisions T1 | Family planning decisions T1 | .037 | .029 | .044 | .030 | .043 |
| Spousal communication T1 | Healthcare decisions T1 | .018 | .011 | .025 | .011 | .024 |
| Spousal communication T1 | Family planning decisions T1 | .012 | .003 | .021 | .004 | .019 |
| Healthcare decisions T1 | Family planning decisions T1 | .056 | .046 | .066 | .048 | .064 |
| Child sex (male=1) | Child age (months) | -.027 | -.027 | -.027 | -.027 | -.027 |
| Child sex (male=1) | Maternal age (years) | -.120 | -.120 | -.120 | -.120 | -.120 |
| Child sex (male=1) | Maternal height (cm) | -.109 | -.109 | -.109 | -.109 | -.109 |
| Child sex (male=1) | household economic status | -.003 | -.003 | -.003 | -.003 | -.003 |
| Child sex (male=1) | Maternal education (any 0/1) | -.001 | -.001 | -.001 | -.001 | -.001 |
| Child sex (male=1) | Household head education (any 0/1) | .005 | .005 | .005 | .005 | .005 |
| Child age (months) | Maternal age (years) | 1.025 | 1.025 | 1.025 | 1.025 | 1.025 |
| Child age (months) | Maternal height (cm) | .733 | .733 | .733 | .733 | .733 |
| Child age (months) | household economic status | -.047 | -.047 | -.047 | -.047 | -.047 |
| Child age (months) | Maternal education (any 0/1) | -.028 | -.028 | -.028 | -.028 | -.028 |
| Child age (months) | Household head education (any 0/1) | -.042 | -.042 | -.042 | -.042 | -.042 |
| Maternal age (years) | Maternal height (cm) | -.176 | -.176 | -.176 | -.176 | -.176 |
| Maternal age (years) | household economic status | .506 | .506 | .506 | .506 | .506 |
| Maternal age (years) | Maternal education (any 0/1) | -.255 | -.255 | -.255 | -.255 | -.255 |
| Maternal age (years) | Household head education (any 0/1) | -.237 | -.237 | -.237 | -.237 | -.237 |
| Maternal height (cm) | household economic status | .190 | .190 | .190 | .190 | .190 |
| Maternal height (cm) | Maternal education (any 0/1) | .027 | .027 | .027 | .027 | .027 |
| Maternal height (cm) | Household head education (any 0/1) | .008 | .008 | .008 | .008 | .008 |
| Household economic status | Maternal education (any 0/1) | .004 | .004 | .004 | .004 | .004 |
| Household economic status | Household head education (any 0/1) | .035 | .035 | .035 | .035 | .035 |
| Maternal education (any 0/1) | Household head education (any 0/1) | .023 | .023 | .023 | .023 | .023 |
| E-HFP | Child sex (male=1) | -.007 | -.007 | -.007 | -.007 | -.007 |
| E-HFP | Child age (months) | -.023 | -.023 | -.023 | -.023 | -.023 |
| E-HFP | Maternal age (years) | -.136 | -.136 | -.136 | -.136 | -.136 |
| E-HFP | Maternal height (cm) | -.042 | -.042 | -.042 | -.042 | -.042 |
| E-HFP | household economic status | -.034 | -.034 | -.034 | -.034 | -.034 |
| E-HFP | Maternal education (any 0/1) | .002 | .002 | .002 | .002 | .002 |
| E-HFP | Household head education (any 0/1) | -.002 | -.002 | -.002 | -.002 | -.002 |
| **Variances** |  |  |  |  |  |  |
| To buy medication for children T1 |  | .145 | .130 | .159 | .133 | .157 |
| To buy small amounts of food T1 |  | .097 | .086 | .107 | .088 | .105 |
| To buy large amounts of food T1 |  | .157 | .146 | .166 | .148 | .165 |
| To buy clothing for self T1 |  | .051 | .040 | .061 | .042 | .060 |
| To buy medicine for self T1 |  | .080 | .068 | .092 | .070 | .090 |
| To buy toiletries T1 |  | .088 | .076 | .099 | .078 | .097 |
| To buy foods for children T1 |  | .135 | .119 | .151 | .122 | .148 |
| To buy medication for children T2 |  | .055 | .044 | .067 | .046 | .065 |
| To buy small amounts of food T2 |  | .085 | .075 | .096 | .077 | .094 |
| To buy large amounts of food T2 |  | .168 | .158 | .177 | .159 | .175 |
| To buy clothing for self T2 |  | .045 | .035 | .056 | .037 | .054 |
| To buy medicine for self T2 |  | .133 | .120 | .146 | .122 | .144 |
| To buy toiletries T2 |  | .057 | .048 | .065 | .049 | .064 |
| To buy foods for children T2 |  | .138 | .126 | .151 | .128 | .149 |
| Talk about expenses T1 |  | .207 | .176 | .236 | .180 | .232 |
| Talk about prof/ag activities T1 |  | .216 | .188 | .246 | .193 | .240 |
| Talk about domestic activities T1 |  | .209 | .178 | .238 | .184 | .234 |
| Talk about community events T1 |  | .345 | .311 | .378 | .317 | .372 |
| Talk about child's health T1 |  | .154 | .132 | .175 | .136 | .173 |
| Talk about child nutrition T1 |  | .185 | .159 | .208 | .163 | .204 |
| Talk about own health T1 |  | .155 | .135 | .177 | .138 | .173 |
| Talk about expenses T2 |  | .025 | .011 | .039 | .013 | .037 |
| Talk about prof/ag activities T2 |  | .082 | .058 | .108 | .061 | .104 |
| Talk about domestic activities T2 |  | .047 | .025 | .068 | .028 | .064 |
| Talk about community events T2 |  | .121 | .091 | .148 | .096 | .144 |
| Talk about child's health T2 |  | .124 | .106 | .142 | .110 | .139 |
| Talk about child nutrition T2 |  | .111 | .092 | .130 | .095 | .127 |
| Talk about own health T2 |  | .115 | .098 | .134 | .101 | .131 |
| Decisions to seek care when pregnant T1 |  | .133 | .116 | .149 | .119 | .147 |
| Decisions for sick child T1 |  | .141 | .125 | .157 | .127 | .154 |
| Decisions to seek care when pregnant T2 |  | .095 | .077 | .113 | .080 | .110 |
| Decisions for sick child T2 |  | .100 | .081 | .117 | .083 | .115 |
| Decisions to use contraception T1 |  | .066 | .041 | .087 | .045 | .084 |
| Decisions to have another child T1 |  | .165 | .150 | .179 | .153 | .177 |
| Decisions to use contraception T2 |  | .069 | .043 | .089 | .048 | .086 |
| Decisions to have another child T2 |  | .170 | .154 | .184 | .157 | .182 |
| Child sex (male=1) |  | .250 | .250 | .250 | .250 | .250 |
| Child age (months) |  | 7.655 | 7.655 | 7.655 | 7.655 | 7.655 |
| Maternal age (years) |  | 40.716 | 40.716 | 40.716 | 40.716 | 40.716 |
| Maternal height (cm) |  | 39.137 | 39.137 | 39.137 | 39.137 | 39.137 |
| Household economic status |  | 1.530 | 1.530 | 1.530 | 1.530 | 1.530 |
| Maternal education (any 0/1) |  | .061 | .061 | .061 | .061 | .061 |
| Household head education (any 0/1) |  | .096 | .096 | .096 | .096 | .096 |
| ∆Wasting |  | .262 | .234 | .286 | .238 | .281 |
| E-HFP |  | .242 | .242 | .242 | .242 | .242 |
| **Means of observed variables** |  |  |  |  |  |  |
| To buy medication for children T1 |  | .533 | .452 | .616 | .466 | .596 |
| To buy small amounts of food T1 |  | .833 | .780 | .882 | .791 | .873 |
| To buy large amounts of food T1 |  | .399 | .337 | .464 | .345 | .451 |
| To buy clothing for self T1 |  | .747 | .661 | .829 | .672 | .816 |
| To buy medicine for self T1 |  | .635 | .546 | .722 | .566 | .700 |
| To buy toiletries T1 |  | .767 | .696 | .839 | .707 | .824 |
| To buy foods for children T1 |  | .613 | .534 | .689 | .547 | .677 |
| To buy medication for children T2 |  | .652 | .558 | .747 | .575 | .728 |
| To buy small amounts of food T2 |  | .820 | .762 | .877 | .771 | .868 |
| To buy large amounts of food T2 |  | .394 | .322 | .472 | .334 | .462 |
| To buy clothing for self T2 |  | .676 | .580 | .778 | .598 | .757 |
| To buy medicine for self T2 |  | .466 | .368 | .570 | .387 | .546 |
| To buy toiletries T2 |  | .761 | .673 | .847 | .695 | .829 |
| To buy foods for children T2 |  | .461 | .370 | .551 | .389 | .535 |
| Talk about expenses T1 |  | 1.601 | 1.434 | 1.760 | 1.456 | 1.738 |
| Talk about prof/ag activities T1 |  | 1.673 | 1.517 | 1.820 | 1.539 | 1.799 |
| Talk about domestic activities T1 |  | 1.624 | 1.459 | 1.786 | 1.484 | 1.762 |
| Talk about community events T1 |  | 1.430 | 1.272 | 1.578 | 1.302 | 1.554 |
| Talk about child's health T1 |  | 1.860 | 1.730 | 1.983 | 1.752 | 1.963 |
| Talk about child nutrition T1 |  | 1.810 | 1.671 | 1.936 | 1.696 | 1.919 |
| Talk about own health T1 |  | 1.860 | 1.729 | 1.983 | 1.752 | 1.964 |
| Talk about expenses T2 |  | 1.213 | 1.073 | 1.355 | 1.095 | 1.334 |
| Talk about prof/ag activities T2 |  | 1.226 | 1.100 | 1.363 | 1.116 | 1.341 |
| Talk about domestic activities T2 |  | 1.191 | 1.054 | 1.337 | 1.072 | 1.312 |
| Talk about community events T2 |  | 1.140 | 1.008 | 1.271 | 1.030 | 1.252 |
| Talk about child's health T2 |  | 1.385 | 1.281 | 1.494 | 1.299 | 1.475 |
| Talk about child nutrition T2 |  | 1.341 | 1.233 | 1.459 | 1.248 | 1.438 |
| Talk about own health T2 |  | 1.373 | 1.268 | 1.480 | 1.284 | 1.463 |
| Decisions to seek care when pregnant T1 |  | .629 | .494 | .776 | .514 | .752 |
| Decisions for sick child T1 |  | .577 | .439 | .720 | .463 | .697 |
| Decisions to seek care when pregnant T2 |  | .527 | .304 | .736 | .346 | .700 |
| Decisions for sick child T2 |  | .535 | .314 | .737 | .358 | .706 |
| Decisions to use contraception T1 |  | .693 | .540 | .838 | .563 | .817 |
| Decisions to have another child T1 |  | .554 | .446 | .652 | .466 | .638 |
| Decisions to use contraception T2 |  | .379 | .137 | .637 | .175 | .593 |
| Decisions to have another child T2 |  | .360 | .196 | .537 | .217 | .508 |
| ∆Wasting |  | .649 | -.289 | 1.666 | -.116 | 1.463 |
| E-HFP |  | .589 | .589 | .589 | .589 | .589 |
| Child sex (male=1) |  | .507 | .507 | .507 | .507 | .507 |
| Child age (months) |  | 7.310 | 7.310 | 7.310 | 7.310 | 7.310 |
| Maternal age (years) |  | 28.403 | 28.403 | 28.403 | 28.403 | 28.403 |
| Maternal height (cm) |  | 161.265 | 161.265 | 161.265 | 161.265 | 161.265 |
| household economic status |  | -.020 | -.020 | -.020 | -.020 | -.020 |
| Maternal education (any 0/1) |  | .065 | .065 | .065 | .065 | .065 |
| Household head education (any 0/1) |  | .107 | .107 | .107 | .107 | .107 |
| **Direct, indirect, and total effects calculated from the path coefficients in the structural equation models** | | | | | | |
| **Direct effect** |  |  |  |  |  |  |
| of E-HFP (*c*' path) |  | -.056 | -.125 | .016 | -.114 | .004 |
| **Individual indirect effects of E-HFP^1^** |  |  |  |  |  |  |
| via ∆purchasing decisions | (a_pur_b_pur)_ | -.003 | -.011 | .005 | -.010 | .004 |
| via ∆spousal communication | (a_com_b_com_) | -.010 | -.022 | -.001 | -.020 | -.002 |
| via ∆healthcare decisions | (a_hlth_b_hlth_) | -.003 | -.014 | .003 | -.011 | .002 |
| via ∆family planning decisions | (a_fp_b_fp_) | -.003 | -.015 | .003 | -.012 | .002 |
| **Total indirect effect^2^** |  |  |  |  |  |  |
| via ∆4 empowerment domains | (a_pur_b_pur_ + a_com_b_com_ + a_hlth_b_hlth_ +a_fp_b_fp_) | -.019 | -.045 | -.002 | -.039 | -.005 |
| **Total effect of E-HFP^3^** | (c' + a_pur_b_pur_ + a_com_b_com_ + a_hlth_b_hlth_ +a_fp_b_fp_) | -.075 | -.146 | -.003 | -.133 | -.014 |
| **Fit statistics** |  |  |  |  |  |  |
| CFI |  | .954 |  |  |  |  |
| TLI |  | .947 |  |  |  |  |
| RMSEA |  | .035 |  |  |  |  |

^1^Indirect effects for each mediator are calculated from the respective *a* and *b* paths using bootstrapping.

^2^Calculated as the sum of the four indirect effects of the four domains of women’s empowerment.

^3^Calculated as the sum of the direct effect and the total indirect effect.

Note: All coefficients are unstandardized and correspond to the paths depicted in Figure 1. E-HFP=Enhanced-Homestead Food Production, LCI=lower confidence interval (95%), UCI=upper confidence interval (95%), CFI=Comparative Fit Index, TLI=Tucker-Lewis Index, RMSEA=Root Mean Square Error of Approximation

**Supplementary Table 5**: Full results of a structural equation model that simultaneously tests four domains of women’s empowerment as mediators between the E-HFP program and changes in child hemoglobin concentration and summary of direct, indirect, and total effects calculated from these models.

|  |  | β | LCI 95% | UCI 95% | LCI 90% | UCI 90% |
| --- | --- | --- | --- | --- | --- | --- |
| **Structural equation model result^1^** |  |  |  |  |  |  |
| **Structural model** |  |  |  |  |  |  |
| **(left-hand side variable)** | **(right-hand side variables)** |  |  |  |  |  |
| ∆Purchasing decisions (equation 1) | E-HFP (a_pur_ path) | .093 | .041 | .145 | .050 | .137 |
|  | Child sex (male=1) | -.022 | -.074 | .027 | -.063 | .021 |
|  | Child age (months) | .000 | -.009 | .008 | -.008 | .007 |
|  | Maternal age (years) | .001 | -.003 | .005 | -.002 | .004 |
|  | Household economic status | -.012 | -.032 | .008 | -.028 | .004 |
|  | Maternal education (any 0/1) | -.051 | -.155 | .053 | -.138 | .038 |
|  | Household head education (any 0/1) | .015 | -.076 | .108 | -.060 | .095 |
| ∆Spousal communication (equation 2) | E-HFP (a_com_ path) | .149 | .086 | .213 | .097 | .205 |
|  | Child sex (male=1) | -.031 | -.092 | .029 | -.083 | .019 |
|  | Child age (months) | -.008 | -.019 | .003 | -.017 | .002 |
|  | Maternal age (years) | .005 | .000 | .010 | .001 | .009 |
|  | Household economic status | .017 | -.011 | .044 | -.006 | .039 |
|  | Maternal education (any 0/1) | -.030 | -.180 | .120 | -.143 | .100 |
|  | Household head education (any 0/1) | -.030 | -.139 | .077 | -.118 | .063 |
| ∆Healthcare decisions (equation 3) | E-HFP (a_hlth_ path) | .041 | -.017 | .099 | -.007 | .089 |
|  | Child sex (male=1) | .049 | -.012 | .106 | -.002 | .097 |
|  | Child age (months) | -.003 | -.013 | .007 | -.012 | .006 |
|  | Maternal age (years) | -.004 | -.008 | .001 | -.008 | .000 |
|  | household economic status | -.003 | -.027 | .020 | -.023 | .017 |
|  | Maternal education (any 0/1) | .040 | -.072 | .163 | -.055 | .138 |
|  | Household head education (any 0/1) | -.020 | -.112 | .085 | -.101 | .073 |
| ∆Family planning decisions (equation 4) | E-HFP (a_fp_ path) | .047 | -.017 | .111 | -.006 | .100 |
|  | Child sex (male=1) | .030 | -.034 | .089 | -.026 | .079 |
|  | Child age (months) | .000 | -.010 | .011 | -.009 | .009 |
|  | Maternal age (years) | -.003 | -.009 | .002 | -.008 | .001 |
|  | household economic status | -.002 | -.028 | .024 | -.024 | .020 |
|  | Maternal education (any 0/1) | -.010 | -.135 | .124 | -.115 | .098 |
|  | Household head education (any 0/1) | .023 | -.069 | .120 | -.055 | .105 |
| ∆Hemoglobin (equation 5) | ∆Purchasing decisions (b_pur_ path) | -.147 | -.452 | .163 | -.415 | .113 |
|  | ∆Spousal communication (b_com_ path) | .108 | -.134 | .358 | -.083 | .320 |
|  | ∆Healthcare decisions (b_hlth_ path) | -.313 | -.911 | .243 | -.808 | .176 |
|  | ∆Family planning decisions (b_fp_ path) | .064 | -.473 | .744 | -.381 | .594 |
|  | E-HFP (c' path) | .292 | .007 | .561 | .063 | .516 |
|  | Child sex (male=1) | .203 | -.061 | .459 | -.026 | .414 |
|  | Child age (months) | .105 | .055 | .154 | .064 | .147 |
|  | Maternal age (years) | .030 | .009 | .053 | .013 | .049 |
|  | Household economic status | .010 | -.094 | .110 | -.076 | .096 |
|  | Maternal education (any 0/1) | -.147 | -.761 | .499 | -.667 | .392 |
|  | Household head education (any 0/1) | -.234 | -.694 | .225 | -.606 | .147 |
| **Measurement components** |  |  |  |  |  |  |
| Purchasing decisions T1 | To buy medication for children T1 | 1.000 | 1.000 | 1.000 | 1.000 | 1.000 |
|  | To buy small amounts of food T1 | .607 | .552 | .659 | .563 | .651 |
|  | To buy large amounts of food T1 | .785 | .743 | .828 | .750 | .820 |
|  | To buy clothing for self T1 | 1.052 | 1.015 | 1.090 | 1.022 | 1.084 |
|  | To buy medicine for self T1 | 1.053 | 1.021 | 1.086 | 1.026 | 1.079 |
|  | To buy toiletries T1 | .882 | .836 | .928 | .844 | .920 |
|  | To buy foods for children T1 | .970 | .935 | 1.002 | .940 | .999 |
| Purchasing decisions T2 | To buy medication for children T2 | 1.000 | 1.000 | 1.000 | 1.000 | 1.000 |
|  | To buy small amounts of food T2 | .607 | .552 | .659 | .563 | .651 |
|  | To buy large amounts of food T2 | .785 | .743 | .828 | .750 | .820 |
|  | To buy clothing for self T2 | 1.052 | 1.015 | 1.090 | 1.022 | 1.084 |
|  | To buy medicine for self T2 | 1.053 | 1.021 | 1.086 | 1.026 | 1.079 |
|  | To buy toiletries T2 | .882 | .836 | .928 | .844 | .920 |
|  | To buy foods for children T2 | .970 | .935 | 1.002 | .940 | .999 |
| Spousal communication T1 | Talk about expenses T1 | 1.000 | 1.000 | 1.000 | 1.000 | 1.000 |
|  | Talk about prof/ag activities T1 | .916 | .871 | .960 | .878 | .954 |
|  | Talk about domestic activities T1 | .975 | .943 | 1.009 | .947 | 1.003 |
|  | Talk about community events T1 | .921 | .870 | .975 | .879 | .967 |
|  | Talk about child's health T1 | .737 | .676 | .798 | .687 | .790 |
|  | Talk about child nutrition T1 | .788 | .724 | .848 | .737 | .839 |
|  | Talk about own health T1 | .759 | .696 | .818 | .707 | .810 |
| Spousal communication T2 | Talk about expenses T2 | 1.000 | 1.000 | 1.000 | 1.000 | 1.000 |
|  | Talk about prof/ag activities T2 | .916 | .871 | .960 | .878 | .954 |
|  | Talk about domestic activities T2 | .975 | .943 | 1.009 | .947 | 1.003 |
|  | Talk about community events T2 | .921 | .870 | .975 | .879 | .967 |
|  | Talk about child's health T2 | .737 | .676 | .798 | .687 | .790 |
|  | Talk about child nutrition T2 | .788 | .724 | .848 | .737 | .839 |
|  | Talk about own health T2 | .759 | .696 | .818 | .707 | .810 |
| Healthcare decisions T1 | Decisions to seek care when pregnant T1 | 1.000 | 1.000 | 1.000 | 1.000 | 1.000 |
|  | Decisions for sick child T1 | .998 | .919 | 1.077 | .936 | 1.062 |
| Healthcare decisions T2 | Decisions to seek care when pregnant T2 | 1.000 | 1.000 | 1.000 | 1.000 | 1.000 |
|  | Decisions for sick child T2 | .998 | .919 | 1.077 | .936 | 1.062 |
| Family planning decisions T1 | Decisions to use contraception T1 | 1.000 | 1.000 | 1.000 | 1.000 | 1.000 |
|  | Decisions to have another child T1 | .678 | .596 | .758 | .612 | .745 |
| Family planning decisions T2 | Decisions to use contraception T2 | 1.000 | 1.000 | 1.000 | 1.000 | 1.000 |
|  | Decisions to have another child T2 | .678 | .596 | .758 | .612 | .745 |
| **Latent change score components** |  |  |  |  |  |  |
| **Latent change score for purchasing decisions** | |  |  |  |  |  |
| Purchasing decisions T2 | Purchasing decisions T1 | 1.000 | 1.000 | 1.000 | 1.000 | 1.000 |
| ∆Purchasing decisions | Purchasing decisions T2 | 1.000 | 1.000 | 1.000 | 1.000 | 1.000 |
| Purchasing decisions T1 |  | -.034 | -.062 | -.006 | -.057 | -.010 |
| ∆Purchasing decisions |  | .002 | -.106 | .114 | -.082 | .086 |
| Purchasing decisions T1 | Purchasing decisions T1 | .132 | .123 | .143 | .124 | .142 |
| Purchasing decisions T1 | ∆Purchasing decisions | -.113 | -.127 | -.099 | -.125 | -.102 |
| ∆Purchasing decisions | ∆Purchasing decisions | .244 | .220 | .270 | .223 | .266 |
| **Latent change score for spousal communication** | |  |  |  |  |  |
| Spousal communication T2 | Spousal communication T1 | 1.000 | 1.000 | 1.000 | 1.000 | 1.000 |
| ∆Spousal communication | Spousal communication T2 | 1.000 | 1.000 | 1.000 | 1.000 | 1.000 |
| Spousal communication T1 |  | .013 | -.024 | .053 | -.019 | .045 |
| ∆Spousal communication |  | -.178 | -.291 | -.073 | -.269 | -.090 |
| Spousal communication T1 | Spousal communication T1 | .133 | .108 | .164 | .112 | .158 |
| Spousal communication T1 | ∆Spousal communication | -.130 | -.164 | -.103 | -.156 | -.107 |
| ∆Spousal communication | ∆Spousal communication | .384 | .341 | .431 | .347 | .419 |
| **Latent change score for healthcare decisions** | |  |  |  |  |  |
| Healthcare decisions T2 | Healthcare decisions T1 | 1.000 | 1.000 | 1.000 | 1.000 | 1.000 |
| ∆Healthcare decisions | Healthcare decisions T2 | 1.000 | 1.000 | 1.000 | 1.000 | 1.000 |
| Healthcare decisions T1 |  | -.038 | -.084 | .009 | -.075 | .001 |
| ∆Healthcare decisions |  | .174 | .044 | .301 | .072 | .279 |
| Healthcare decisions T1 | Healthcare decisions T1 | .088 | .073 | .104 | .076 | .101 |
| Healthcare decisions T1 | ∆Healthcare decisions | -.054 | -.073 | -.036 | -.068 | -.039 |
| ∆Healthcare decisions | ∆Healthcare decisions | .202 | .172 | .232 | .178 | .226 |
| **Latent change score for family planning decisions** | |  |  |  |  |  |
| Family planning decisions T2 | Family planning decisions T1 | 1.000 | 1.000 | 1.000 | 1.000 | 1.000 |
| ∆Family planning decisions | Family planning decisions T2 | 1.000 | 1.000 | 1.000 | 1.000 | 1.000 |
| Family planning decisions T1 |  | -.013 | -.051 | .028 | -.044 | .021 |
| ∆Family planning decisions |  | .083 | -.011 | .177 | .001 | .160 |
| Family planning decisions T1 | Family planning decisions T1 | .149 | .126 | .174 | .130 | .169 |
| Family planning decisions T1 | ∆Family planning decisions | -.078 | -.109 | -.051 | -.104 | -.055 |
| ∆Family planning decisions | ∆Family planning decisions | .227 | .180 | .276 | .186 | .267 |
| **Observed characteristics with correlated error terms** | |  |  |  |  |  |
| To buy small amounts of food T1 | To buy toiletries T1 | -.020 | -.029 | -.011 | -.027 | -.012 |
| To buy large amounts of food T1 | To buy clothing for self T1 | -.012 | -.020 | -.005 | -.018 | -.005 |
| To buy large amounts of food T1 | To buy medicine for self T1 | .014 | .004 | .025 | .006 | .023 |
| To buy large amounts of food T1 | To buy foods for children T1 | .032 | .021 | .044 | .023 | .041 |
| To buy clothing for self T1 | To buy foods for children T1 | -.018 | -.025 | -.010 | -.024 | -.011 |
| To buy medication for children T1 | To buy large amounts of food T1 | .047 | .035 | .060 | .037 | .058 |
| To buy medication for children T1 | To buy medicine for self T1 | .053 | .043 | .063 | .044 | .061 |
| To buy medication for children T1 | To buy toiletries T1 | .009 | .002 | .015 | .003 | .014 |
| To buy medication for children T1 | To buy foods for children T1 | .073 | .061 | .086 | .063 | .084 |
| To buy small amounts of food T2 | To buy toiletries T2 | .021 | .014 | .028 | .015 | .027 |
| To buy large amounts of food T2 | To buy foods for children T2 | .052 | .040 | .064 | .042 | .062 |
| To buy large amounts of food T2 | To buy medicine for self T2 | .048 | .036 | .059 | .038 | .058 |
| To buy clothing for self T2 | To buy foods for children T2 | -.006 | -.012 | .000 | -.011 | -.001 |
| To buy medicine for self T2 | To buy foods for children T2 | .099 | .086 | .112 | .088 | .110 |
| To buy medication for children T2 | To buy clothing for self T2 | .009 | .000 | .017 | .002 | .016 |
| To buy medication for children T2 | To buy foods for children T2 | .016 | .009 | .023 | .010 | .021 |
| Talk about expenses T1 | Talk about domestic activities T1 | .107 | .078 | .135 | .082 | .131 |
| Talk about prof/ag activities T1 | Talk about domestic activities T1 | .082 | .055 | .110 | .059 | .105 |
| Talk about child's health T1 | Talk about own health T1 | .092 | .074 | .109 | .077 | .106 |
| Talk about prof/ag activities T1 | Talk about community events T1 | .112 | .085 | .142 | .089 | .136 |
| Talk about child nutrition T1 | Talk about own health T1 | .088 | .068 | .107 | .072 | .103 |
| Talk about community events T1 | Talk about own health T1 | .013 | .000 | .025 | .002 | .023 |
| Talk about child's health T1 | Talk about child nutrition T1 | .079 | .061 | .098 | .065 | .094 |
| Talk about prof/ag activities T1 | Talk about child's health T1 | -.020 | -.033 | -.009 | -.031 | -.011 |
| Talk about expenses T1 | Talk about community events T1 | .105 | .076 | .134 | .079 | .130 |
| Talk about domestic activities T1 | Talk about community events T1 | .103 | .074 | .133 | .078 | .128 |
| Talk about expenses T1 | Talk about prof/ag activities T1 | .057 | .030 | .084 | .035 | .080 |
| Talk about expenses T1 | Talk about child nutrition T1 | .013 | .002 | .024 | .004 | .022 |
| Talk about expenses T2 | Talk about domestic activities T2 | -.010 | -.024 | .006 | -.022 | .004 |
| Talk about child's health T2 | Talk about own health T2 | .095 | .078 | .111 | .081 | .108 |
| Talk about prof/ag activities T2 | Talk about domestic activities T2 | .004 | -.010 | .018 | -.008 | .016 |
| Talk about child nutrition T2 | Talk about own health T2 | .083 | .066 | .100 | .069 | .098 |
| Talk about child's health T2 | Talk about child nutrition T2 | .080 | .064 | .097 | .066 | .094 |
| Talk about expenses T2 | Talk about prof/ag activities T2 | -.017 | -.029 | -.007 | -.026 | -.008 |
| **Correlations between mediators** |  |  |  |  |  |  |
| ∆Purchasing decisions | ∆Spousal communication | .015 | .002 | .029 | .003 | .026 |
| ∆Purchasing decisions | ∆Healthcare decisions | .052 | .039 | .063 | .041 | .062 |
| ∆Spousal communication | ∆Healthcare decisions | -.025 | -.041 | -.009 | -.039 | -.011 |
| ∆Purchasing decisions | ∆Family planning decisions | .027 | .014 | .040 | .016 | .038 |
| ∆Spousal communication | ∆Family planning decisions | .018 | .001 | .035 | .004 | .032 |
| ∆Healthcare decisions | ∆Family planning decisions | .100 | .083 | .116 | .086 | .113 |
| **Correlations among T1 variables (excluding measurement model)** | |  |  |  |  |  |
| Purchasing decisions T1 | Spousal communication T1 | .015 | .008 | .022 | .009 | .021 |
| Purchasing decisions T1 | Healthcare decisions T1 | .026 | .019 | .033 | .020 | .032 |
| Purchasing decisions T1 | Family planning decisions T1 | .037 | .029 | .044 | .030 | .043 |
| Spousal communication T1 | Healthcare decisions T1 | .018 | .010 | .025 | .012 | .024 |
| Spousal communication T1 | Family planning decisions T1 | .012 | .003 | .021 | .004 | .020 |
| Healthcare decisions T1 | Family planning decisions T1 | .056 | .047 | .065 | .048 | .064 |
| Child sex (male=1) | Child age (months) | -.027 | -.027 | -.027 | -.027 | -.027 |
| Child sex (male=1) | Maternal age (years) | -.120 | -.120 | -.120 | -.120 | -.120 |
| Child sex (male=1) | household economic status | -.003 | -.003 | -.003 | -.003 | -.003 |
| Child sex (male=1) | Maternal education (any 0/1) | -.001 | -.001 | -.001 | -.001 | -.001 |
| Child sex (male=1) | Household head education (any 0/1) | .005 | .005 | .005 | .005 | .005 |
| Child age (months) | Maternal age (years) | 1.024 | 1.024 | 1.024 | 1.024 | 1.024 |
| Child age (months) | household economic status | -.047 | -.047 | -.047 | -.047 | -.047 |
| Child age (months) | Maternal education (any 0/1) | -.028 | -.028 | -.028 | -.028 | -.028 |
| Child age (months) | Household head education (any 0/1) | -.041 | -.041 | -.041 | -.041 | -.041 |
| Maternal age (years) | household economic status | .503 | .503 | .503 | .503 | .503 |
| Maternal age (years) | Maternal education (any 0/1) | -.253 | -.253 | -.253 | -.253 | -.253 |
| Maternal age (years) | Household head education (any 0/1) | -.236 | -.236 | -.236 | -.236 | -.236 |
| Household economic status | Maternal education (any 0/1) | .004 | .004 | .004 | .004 | .004 |
| Household economic status | Household head education (any 0/1) | .036 | .036 | .036 | .036 | .036 |
| Maternal education (any 0/1) | Household head education (any 0/1) | .023 | .023 | .023 | .023 | .023 |
| E-HFP | Child sex (male=1) | -.007 | -.007 | -.007 | -.007 | -.007 |
| E-HFP | Child age (months) | -.023 | -.023 | -.023 | -.023 | -.023 |
| E-HFP | Maternal age (years) | -.136 | -.136 | -.136 | -.136 | -.136 |
| E-HFP | household economic status | -.034 | -.034 | -.034 | -.034 | -.034 |
| E-HFP | Maternal education (any 0/1) | .002 | .002 | .002 | .002 | .002 |
| E-HFP | Household head education (any 0/1) | -.002 | -.002 | -.002 | -.002 | -.002 |
| **Variances** |  |  |  |  |  |  |
| To buy medication for children T1 |  | .145 | .130 | .159 | .133 | .157 |
| To buy small amounts of food T1 |  | .097 | .086 | .107 | .087 | .105 |
| To buy large amounts of food T1 |  | .157 | .147 | .167 | .148 | .166 |
| To buy clothing for self T1 |  | .051 | .041 | .063 | .042 | .060 |
| To buy medicine for self T1 |  | .080 | .068 | .092 | .069 | .090 |
| To buy toiletries T1 |  | .088 | .076 | .099 | .078 | .097 |
| To buy foods for children T1 |  | .135 | .121 | .151 | .123 | .148 |
| To buy medication for children T2 |  | .055 | .044 | .066 | .046 | .064 |
| To buy small amounts of food T2 |  | .085 | .075 | .095 | .077 | .094 |
| To buy large amounts of food T2 |  | .168 | .157 | .177 | .159 | .176 |
| To buy clothing for self T2 |  | .045 | .035 | .055 | .036 | .053 |
| To buy medicine for self T2 |  | .133 | .120 | .146 | .122 | .144 |
| To buy toiletries T2 |  | .057 | .047 | .066 | .049 | .064 |
| To buy foods for children T2 |  | .138 | .125 | .152 | .127 | .149 |
| Talk about expenses T1 |  | .207 | .176 | .238 | .180 | .234 |
| Talk about prof/ag activities T1 |  | .216 | .188 | .243 | .192 | .239 |
| Talk about domestic activities T1 |  | .209 | .179 | .239 | .183 | .236 |
| Talk about community events T1 |  | .346 | .311 | .382 | .316 | .376 |
| Talk about child's health T1 |  | .154 | .133 | .175 | .136 | .173 |
| Talk about child nutrition T1 |  | .184 | .160 | .209 | .163 | .205 |
| Talk about own health T1 |  | .155 | .133 | .175 | .136 | .172 |
| Talk about expenses T2 |  | .025 | .010 | .041 | .012 | .038 |
| Talk about prof/ag activities T2 |  | .082 | .058 | .106 | .062 | .102 |
| Talk about domestic activities T2 |  | .047 | .026 | .069 | .029 | .065 |
| Talk about community events T2 |  | .121 | .091 | .150 | .095 | .145 |
| Talk about child's health T2 |  | .123 | .106 | .140 | .109 | .137 |
| Talk about child nutrition T2 |  | .110 | .093 | .130 | .095 | .125 |
| Talk about own health T2 |  | .115 | .098 | .133 | .101 | .130 |
| Decisions to seek care when pregnant T1 |  | .133 | .115 | .149 | .118 | .146 |
| Decisions for sick child T1 |  | .140 | .125 | .155 | .127 | .153 |
| Decisions to seek care when pregnant T2 |  | .095 | .077 | .112 | .081 | .110 |
| Decisions for sick child T2 |  | .100 | .082 | .118 | .085 | .115 |
| Decisions to use contraception T1 |  | .066 | .040 | .090 | .045 | .086 |
| Decisions to have another child T1 |  | .165 | .149 | .178 | .152 | .176 |
| Decisions to use contraception T2 |  | .069 | .046 | .090 | .049 | .087 |
| Decisions to have another child T2 |  | .170 | .154 | .186 | .157 | .183 |
| Child sex (male=1) |  | .250 | .250 | .250 | .250 | .250 |
| Child age (months) |  | 7.655 | 7.655 | 7.655 | 7.655 | 7.655 |
| Maternal age (years) |  | 40.711 | 40.711 | 40.711 | 40.711 | 40.711 |
| Household economic status |  | 1.530 | 1.530 | 1.530 | 1.530 | 1.530 |
| Maternal education (any 0/1) |  | .061 | .061 | .061 | .061 | .061 |
| Household head education (any 0/1) |  | .096 | .096 | .096 | .096 | .096 |
| ∆Hemoglobin |  | 4.495 | 4.069 | 4.855 | 4.112 | 4.792 |
| E-HFP |  | .242 | .242 | .242 | .242 | .242 |
| **Means of observed variables** |  |  |  |  |  |  |
| To buy medication for children T1 |  | .538 | .515 | .564 | .518 | .559 |
| To buy small amounts of food T1 |  | .836 | .814 | .859 | .818 | .855 |
| To buy large amounts of food T1 |  | .403 | .377 | .430 | .381 | .426 |
| To buy clothing for self T1 |  | .752 | .727 | .777 | .731 | .772 |
| To buy medicine for self T1 |  | .640 | .616 | .668 | .620 | .662 |
| To buy toiletries T1 |  | .771 | .747 | .796 | .750 | .792 |
| To buy foods for children T1 |  | .617 | .591 | .643 | .596 | .639 |
| To buy medication for children T2 |  | .643 | .616 | .669 | .620 | .664 |
| To buy small amounts of food T2 |  | .814 | .791 | .837 | .794 | .834 |
| To buy large amounts of food T2 |  | .386 | .360 | .413 | .364 | .410 |
| To buy clothing for self T2 |  | .666 | .638 | .693 | .642 | .688 |
| To buy medicine for self T2 |  | .456 | .428 | .483 | .432 | .479 |
| To buy toiletries T2 |  | .752 | .726 | .777 | .730 | .773 |
| To buy foods for children T2 |  | .452 | .423 | .479 | .428 | .474 |
| Talk about expenses T1 |  | 1.440 | 1.399 | 1.481 | 1.407 | 1.475 |
| Talk about prof/ag activities T1 |  | 1.526 | 1.488 | 1.567 | 1.495 | 1.559 |
| Talk about domestic activities T1 |  | 1.467 | 1.424 | 1.512 | 1.432 | 1.504 |
| Talk about community events T1 |  | 1.283 | 1.240 | 1.328 | 1.246 | 1.319 |
| Talk about child's health T1 |  | 1.742 | 1.706 | 1.776 | 1.712 | 1.773 |
| Talk about child nutrition T1 |  | 1.683 | 1.646 | 1.724 | 1.652 | 1.717 |
| Talk about own health T1 |  | 1.738 | 1.701 | 1.773 | 1.709 | 1.768 |
| Talk about expenses T2 |  | 1.410 | 1.364 | 1.457 | 1.370 | 1.449 |
| Talk about prof/ag activities T2 |  | 1.407 | 1.364 | 1.450 | 1.371 | 1.443 |
| Talk about domestic activities T2 |  | 1.383 | 1.338 | 1.429 | 1.344 | 1.421 |
| Talk about community events T2 |  | 1.322 | 1.277 | 1.366 | 1.282 | 1.358 |
| Talk about child's health T2 |  | 1.531 | 1.491 | 1.568 | 1.499 | 1.562 |
| Talk about child nutrition T2 |  | 1.496 | 1.456 | 1.535 | 1.463 | 1.527 |
| Talk about own health T2 |  | 1.522 | 1.483 | 1.559 | 1.489 | 1.552 |
| Decisions to seek care when pregnant T1 |  | .684 | .636 | .732 | .643 | .726 |
| Decisions for sick child T1 |  | .633 | .582 | .682 | .589 | .673 |
| Decisions to seek care when pregnant T2 |  | .442 | .375 | .512 | .384 | .499 |
| Decisions for sick child T2 |  | .450 | .381 | .521 | .393 | .511 |
| Decisions to use contraception T1 |  | .653 | .614 | .694 | .621 | .688 |
| Decisions to have another child T1 |  | .527 | .492 | .564 | .498 | .557 |
| Decisions to use contraception T2 |  | .445 | .381 | .512 | .391 | .497 |
| Decisions to have another child T2 |  | .405 | .356 | .448 | .367 | .440 |
| ∆Hemoglobin |  | -1.076 | -1.796 | -.406 | -1.649 | -.531 |
| E-HFP |  | .589 | .589 | .589 | .589 | .589 |
| Child sex (male=1) |  | .507 | .507 | .507 | .507 | .507 |
| Child age (months) |  | 7.310 | 7.310 | 7.310 | 7.310 | 7.310 |
| Maternal age (years) |  | 28.405 | 28.405 | 28.405 | 28.405 | 28.405 |
| household economic status |  | -.020 | -.020 | -.020 | -.020 | -.020 |
| Maternal education (any 0/1) |  | .065 | .065 | .065 | .065 | .065 |
| Household head education (any 0/1) |  | .107 | .107 | .107 | .107 | .107 |
| **Direct, indirect, and total effects calculated from the path coefficients in the structural equation models** | | | | | | |
| **Direct effect** |  |  |  |  |  |  |
| of E-HFP (*c*' path) |  | .292 | .007 | .561 | .063 | .516 |
| **Individual indirect effects of E-HFP^1^** |  |  |  |  |  |  |
| via ∆purchasing decisions | (a_pur_b_pur)_ | -.014 | -.049 | .016 | -.042 | .010 |
| via ∆spousal communication | (a_com_b_com_) | .016 | -.020 | .058 | -.013 | .050 |
| via ∆healthcare decisions | (a_hlth_b_hlth_) | -.013 | -.054 | .017 | -.047 | .011 |
| via ∆family planning decisions | (a_fp_b_fp_) | -.015 | -.061 | .017 | -.047 | .010 |
| **Total indirect effect^2^** |  |  |  |  |  |  |
| via ∆4 empowerment domains | (a_pur_b_pur_ + a_com_b_com_ + a_hlth_b_hlth_ +a_fp_b_fp_) | -.025 | -.119 | .052 | -.095 | .038 |
| **Total effect of E-HFP^3^** | (c' + a_pur_*b_pur_ + a_com_b_com_ + a_hlth_b_hlth_ +a_fp_b_fp_) | .266 | -.004 | .535 | .029 | .492 |
| **Fit statistics** |  |  |  |  |  |  |
| CFI |  | .954 |  |  |  |  |
| TLI |  | .947 |  |  |  |  |
| RMSEA |  | .036 |  |  |  |  |

^1^Indirect effects for each mediator are calculated from the respective *a* and *b* paths using bootstrapping.

^2^Calculated as the sum of the four indirect effects of the four domains of women’s empowerment.

^3^Calculated as the sum of the direct effect and the total indirect effect.

Note: All coefficients are unstandardized and correspond to the paths depicted in Figure 1. E-HFP=Enhanced-Homestead Food Production, LCI=lower confidence interval (95%), UCI=upper confidence interval (95%), CFI=Comparative Fit Index, TLI=Tucker-Lewis Index, RMSEA=Root Mean Square Error of Approximation
